# Artificial Protein Cage with Unusual Geometry and Regularly Embedded Gold Nanoparticles

**DOI:** 10.1021/acs.nanolett.1c04222

**Published:** 2022-03-07

**Authors:** Karolina Majsterkiewicz, Artur P. Biela, Sourav Maity, Mohit Sharma, Bernard M. A. G. Piette, Agnieszka Kowalczyk, Szymon Gaweł, Soumyananda Chakraborti, Wouter H. Roos, Jonathan G. Heddle

**Affiliations:** † Małopolska Centre of Biotechnology, Jagiellonian University, Kraków 30-387, Poland; ‡ Postgraduate School of Molecular Medicine, ul. Żwirki i Wigury 61, Warsaw 02-091, Poland; § Institute of Zoology and Biomedical Research, Department of Cell Biology and Imaging, Jagiellonian University, Kraków 30-387, Poland; ∥ Moleculaire Biofysica, Zernike Instituut, Rijksuniversiteit Groningen, Groningen 9747 AG, Netherlands; ⊥ Department for Mathematical Sciences, Durham University, Durham DH1 3LE, United Kingdom; # Faculty of Mathematics and Computer Science, Jagiellonian University, Kraków 30-348, Poland

**Keywords:** protein nanocage, protein engineering, gold nanoparticles, nanobiology, programmable proteins, gold nanoparticle arrays

## Abstract

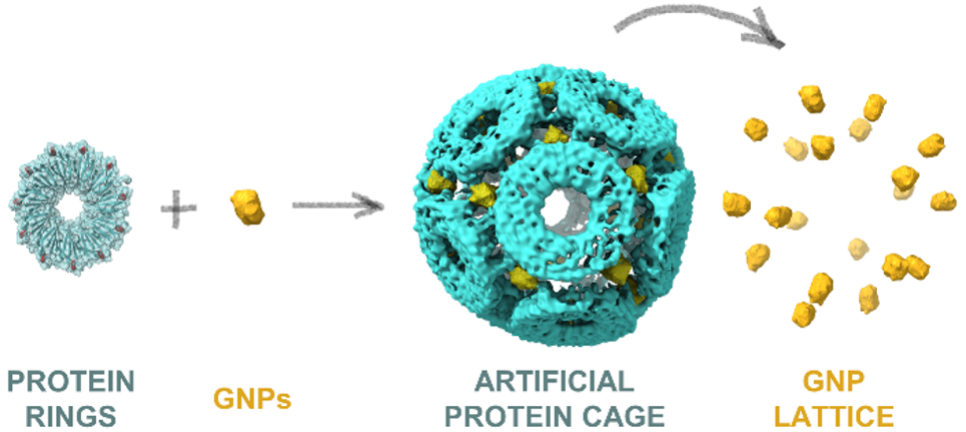

Artificial protein cages have great potential in a number of areas including cargo capture and delivery and as artificial vaccines. Here, we investigate an artificial protein cage whose assembly is triggered by gold nanoparticles. Using biochemical and biophysical methods we were able to determine both the mechanical properties and the gross compositional features of the cage which, combined with mathematical models and biophysical data, allowed the structure of the cage to be predicted. The accuracy of the overall geometrical prediction was confirmed by the cryo-EM structure determined to sub-5 Å resolution. This showed the cage to be nonregular but similar to a dodecahedron, being constructed from 12 11-membered rings. Surprisingly, the structure revealed that the cage also contained a single, small gold nanoparticle at each 3-fold axis meaning that each cage acts as a synthetic framework for regular arrangement of 20 gold nanoparticles in a three-dimensional lattice.

## Introduction

In nature, protein cages provide a number of useful functions such as protective storage of potentially harmful materials (ferritin^1^) and delivery of cargos to cells (viruses^2^). A goal of synthetic biology is to design and produce artificial equivalents of such structures with features that improve or extend those accessible in nature.

TRAP-cage is an artificial protein cage made from trp RNA-binding attenuation protein (TRAP), a ring-shaped bacterial protein made from 11 identical monomers.^3−5^ As well as being characterized structurally and biochemically,^3,4,6−8^ it has also been used as a component of prototype electronics,^9^ a building block for nanotubes,^10,11^ and artificial protein cages (TRAP-cages).^12−14^ The latter are formed using an engineered TRAP produced by a replacement of lysine at position 35 with a cysteine, giving 11 cysteines per ring (TRAP has no native cysteines). Typically, a second mutation of arginine at position 64 to serine is used to decrease nonspecific interactions with gold nanoparticles (GNPs) (Figure S1a–c). The resulting mutant TRAP ring is hereafter referred to as TRAP^CS^.

Two forms of TRAP-cage, large and small, have been identified, and both can be formed by incubation of TRAP^CS^ with GNPs. The approximately 22 nm diameter larger cage (TRAP-LC^GNP^) is formed from 24 TRAP rings, recently suggested to be the most energetically favorable given a hendecamer building block.^15^ The large TRAP-cage is highly stable^14^ and is held together not by noncovalent interactions spanning protein–protein interfaces, but rather almost exclusively by coordinate bonds where single Au(I) atoms bridge two sulfur atoms from opposing cysteine side chains (Figure S1c). As a result, its disassembly can be triggered by reagents containing free thiols, able to etch the gold atom.^14^ The second form of TRAP-cage formed in the presence of GNPs (TRAP-SC^GNP^) is smaller, being 16 nm in diameter.^13^

The ability to arrange and control arrays of metal nanoparticles in two- or three-dimensions is desirable due to the unusual properties they often exhibit, which can be tuned by their arrangement and spacing.^16^ GNPs themselves have interesting properties, larger particles (>approximately 5 nm)^17^ exhibit plasmonic effects while smaller particles are catalytically active. Arrayed gold nanoparticles have shown particularly interesting plasmonic properties^18^ and efforts to control their ordered arrangements have continued.^19^

In contrast to the large TRAP-cage, the smaller cage is completely uncharacterized but poses a number of interesting questions of its own. Its smaller size necessitates that it should be constructed from fewer rings than the large cage but as it is still forbidden from forming a regular-faced convex polyhedron, exactly what shape is formed? Given that a 24-ring cage is the most energetically favored arrangement for a hendecamer, a smaller cage with fewer rings may be expected to be less stable and have a different way of overcoming geometrical restrictions. Both large and small cages are also uncharacterized with respect to their mechanical properties. For interaction with and delivery to cells, these are of particular importance.

In this work, we produced and characterized TRAP-SC^GNP^, including mechanical and structural determination, which we find to be thermally and chemically stable and pH resistant but to a different extent than TRAP-LC^GNP^. The cryo-EM structure of TRAP-SC^GNP^ shows that it consists of 12 TRAP rings arranged to approximate a dodecahedron, an unexpected arrangement for hendecamers with the single Au(I) “crosslinkers” being replaced by gold “staples” consisting of several gold atoms. Perhaps most surprisingly, TRAP-SC^GNP^ acts as a scaffold for three-dimensional GNP lattice formation with each GNP being placed at the equivalent of the vertex of a dodecahedron.

## Assembly of TRAP-SC^GNP^

**Figure 1:**
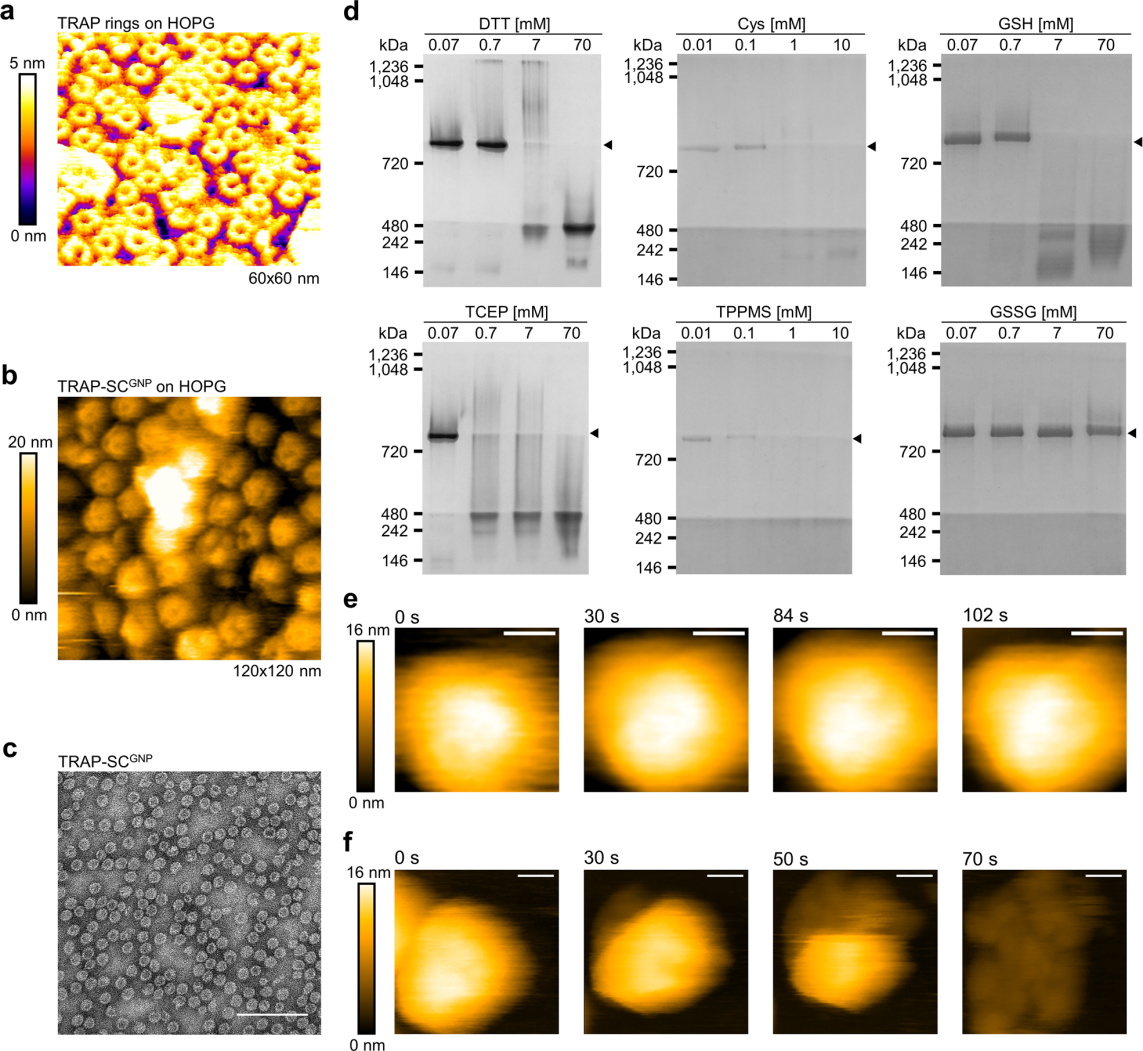
TRAP-SC^GNP^ characterization and triggered disassembly. (a) TRAP(K35C/R64S) rings visualized by AFM (3D rendered, top view). (b) TRAP-SC^GNP^ visualized by AFM (3D rendered, top view). (c) Electron micrograph of purified TRAP-SC^GNP^ (scale bar = 100 nm). (d) Controlled disassembly of TRAP-SC^GNP^ in the presence of compounds containing thiol or phosphine groups: dithiothreitol (DTT), tris(2-carboxyethyl)phosphine (TCEP), l-cysteine (Cys), sodium salt of 3-(diphenylphosphino)benzenesulfonic acid (TPPMS), and reduced or oxidized glutathione (GSH and GSSG, respectively). On the gels, arrowheads indicate the position of small cage. (e) Frames from a HS-AFM movie (Movie S1) of TRAP-SC^GNP^ in absence of DTT (frame rate 0.5 fps, scale bar = 10 nm). (f) Frames from a HS-AFM movie (Movie S2) of TRAP-SC^GNP^ in the presence of 3 mM DTT (frame rate 0.5 fps, scale bar = 10 nm).

TRAP-SC^GNP^ was initially identified as one component of a mix of at least two differently sized TRAP-cages formed by incubation with GNPs in simple aqueous reaction buffer.^13^ By careful titration and consistent with earlier results,^13^ we were able to optimize conditions where the small cage was formed as the majority (Figure S1d). Contaminants were removed by size exclusion chromatography (Figure S1e–g), and the purity of the sample was monitored by blue native PAGE with TRAP-SC^GNP^ forming a clear band and predictably migrating faster than the larger TRAP-cage (Figure S1g). The identity of the TRAP-SC^GNP^ was confirmed by atomic force microscopy (AFM), where the small cage is clearly distinct from unreacted TRAP rings (Figure 1a,b). Transmission electron microscopy (TEM) images also revealed intact spherical structures (Figure 1c). We additionally observed that that purified samples of TRAP-SC^GNP^ retain a slight brownish hue suggesting the presence of the GNPs.

## Stability and Disassembly of TRAP-SC^GNP^

Stability tests (Figure S2) of TRAP-SC^GNP^ showed noticeable aggregation at 50–60 °C and some disassembly at 80 °C, though TEM imaging of samples suggested the majority remain as intact cages even after 10 min at 90 °C (Figure S2a). While high, this thermostability is lower than TRAP-LC^GNP^ which was less aggregation-prone and which exhibited less disassembly products even after 180 min at 95 °C.^14^ TRAP-SC^GNP^ showed stability over pHs 4–11 (Figure S2c) compared to 3–12 for a large cage.^14^ Further comparison of TRAP-LC^GNP^ and TRAP-SC^GNP^ showed similar stability in the presence of SDS, however other chaotropic agents disrupted the small cage more easily with a threshold for guanidinium hydrochloride around 2 M and urea–around 3 M (Figure S2d–e).

Tests with agents containing thiol or phosphine groups (DTT, TCEP, GSH, Cys, TPPMS) revealed that, as expected, they could trigger cage disassembly at comparable concentrations while the oxidized form of glutathione left the structures of both cages intact (Figure 1d, Figure S3). Monitoring TRAP-SC^GNP^ disassembly in the absence and presence of DTT by high-speed AFM (HS-AFM)^12,20^ allowed dynamic observation of the disassembly of TRAP-SC^GNP^ upon addition of a reducing agent (Figure 1e,f, Movies S1 and S2). Overall TRAP-SC^GNP^ appears highly stable (see Table S1 for summary) implying a structural role for gold as a cross-linker between TRAP rings in the small cage similar to that shown for the large cage (Figure S1b,c).^14^

## Mechanical properties of TRAP-SC^GNP^ and TRAP-LC^GNP^

To date, mechanical stability of a number of viruses/virus-like particles has been determined experimentally.^21^ Results show that different viruses have different softness/rigidity and this property can, for instance, depend on the presence or absence of nucleic acids inside the virus capsid and on environmental conditions.^22,23^ Mechanical properties can also influence the propensity for genome uncoating of the protein cage particles after entry into the cell.^24^

While both TRAP-LC^GNP^ and TRAP-SC^GNP^ are highly chemically and thermally stable,^14^ the mechanical properties of neither has been assessed.

**Figure 2:**
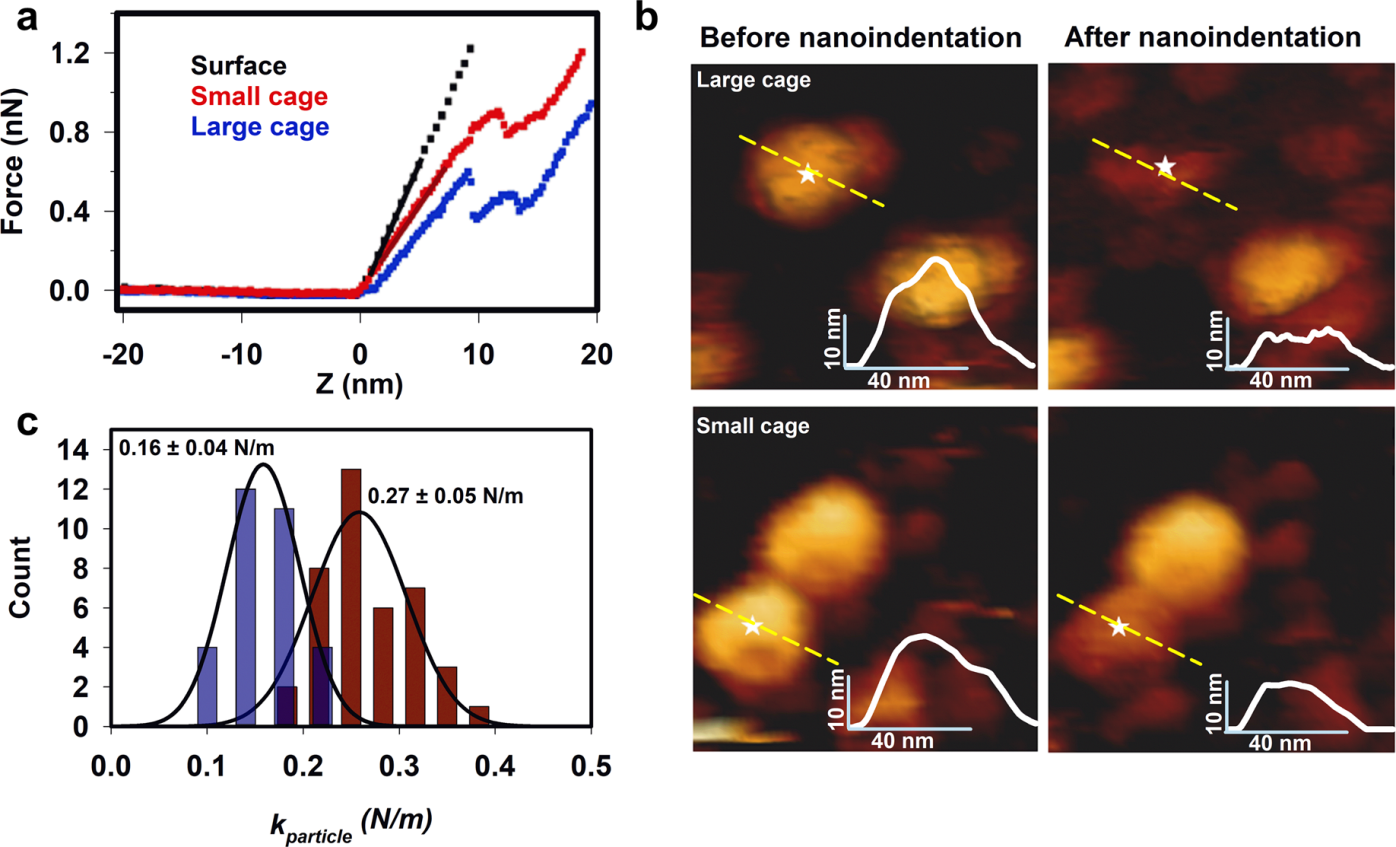
Mechanical properties of TRAP-SC^GNP^ and comparison with TRAP-LC^GNP^. (a) Example of AFM F-D curves taken by pushing on the surface (black), on a TRAP-SC^GNP^ (red), and on a TRAP-LC^GNP^ (blue). (b) Image taken before and after the nanoindentation. The inset shows the height profile of the cross section along the white dotted line in the corresponding image. (c) Histogram of the measured spring constants (*k*_particle_) of TRAP-SC^GNP^ (red) and TRAP-LC^GNP^ (blue).

AFM-based nanoindentation experiments^21,25^ for both TRAP-LC^GNP^ and TRAP-SC^GNP^ cage showed no observable difference in breaking force, indicating similar brittleness. TRAP-SC^GNP^ shows a higher stiffness of 0.27 ± 0.05 N/m with respect to TRAP-LC^GNP^ with a stiffness of 0.16 ± 0.05 N/m (Figure 2). This is not unexpected for a small cage consisting of the same type of subunits as the large cage. The absolute thickness of the protein shell in the small cage is identical to that in the large cage, while the latter has a larger diameter. Thus, the small cage has a relatively thicker shell leading to a higher stiffness. This effect has previously also been shown for the dimorphic hepatitis B virus capsids.^26,27^ To obtain an estimate of the intrinsic material properties of the shell, typically the Young’s modulus is calculated, which is a geometry independent material parameter. Using a thin-shell theory approach^28^ and considering an outer diameter of 17 and 22 nm for small and large cage, respectively, and a shell thickness of 3.5 nm for both results in a Young’s modulus of ∼150 and ∼120 MPa, respectively. Therefore, although the spring constants are almost a factor of 2 apart, the Young’s modulus of both particles is approximately similar. It indicates that the mechanical properties of the material of which the cages are constructed are comparable for both cages, falling in the range of soft viral particles.^29^

## Structural Features of TRAP-SC^GNP^

We employed multiple approaches to gain insight into the size of TRAP-SC^GNP^ and the number of rings present in the structure. Dynamic light scattering (DLS, Figure S4a) measurements indicated a mean diameter of (18.1 ± 0.2) nm with a very low polydispersity index of 0.014. TEM analysis suggested a mean diameter of (16 ± 1) nm consistent with previous results (Figure S4b).^13^

Running the protein on a native gel results in a corresponding band slightly above the 720 kDa marker, while TRAP-LC^GNP^ (MW approximately 2220 kDa) runs close to the 1048 kDa marker (Figure S1g). Simplistically, this suggests that the real MW is approximately double that observed on native PAGE, giving an estimate of 1.5 MDa for TRAP-SC^GNP^. However, native PAGE is not a reliable guide to real MW. For a more accurate measurement, we used size exclusion chromatography (SEC) with right-angle light scattering/low-angle light scattering (RALS/LALS) detection which resulted in a mean value of (1.22 ± 0.03) MDa (mean value from four measurements; exemplary measurement presented on Figure S4c). Considering an individual TRAP ring molecular weight of ∼92 kDa (assuming saturation with tryptophan ligand), a mass of 1.22 MDa would correspond to 13 or 14 TRAP rings. *M*_W_/*M*_N_ (weight-average to number-average molecular weight) ratio for mean molecular weights equals 1.003 indicating that all samples used for measurements were highly monodisperse (for an ideal monodisperse solution this ratio should be 1). The *M*_W_ estimate is likely to be an upper limit given that GNPs may also be present in the structure.

More structural details were obtained by performing HS-AFM image analysis (Figure S4d). The ring diameter, small cage height, and dihedral angle between rings were determined to be (7.9 ± 0.4) nm, (17 ± 1) nm, and (122 ± 4)° respectively (Figure S5).

## Predicting TRAP-SC^GNP^ structure

**Figure 3:**
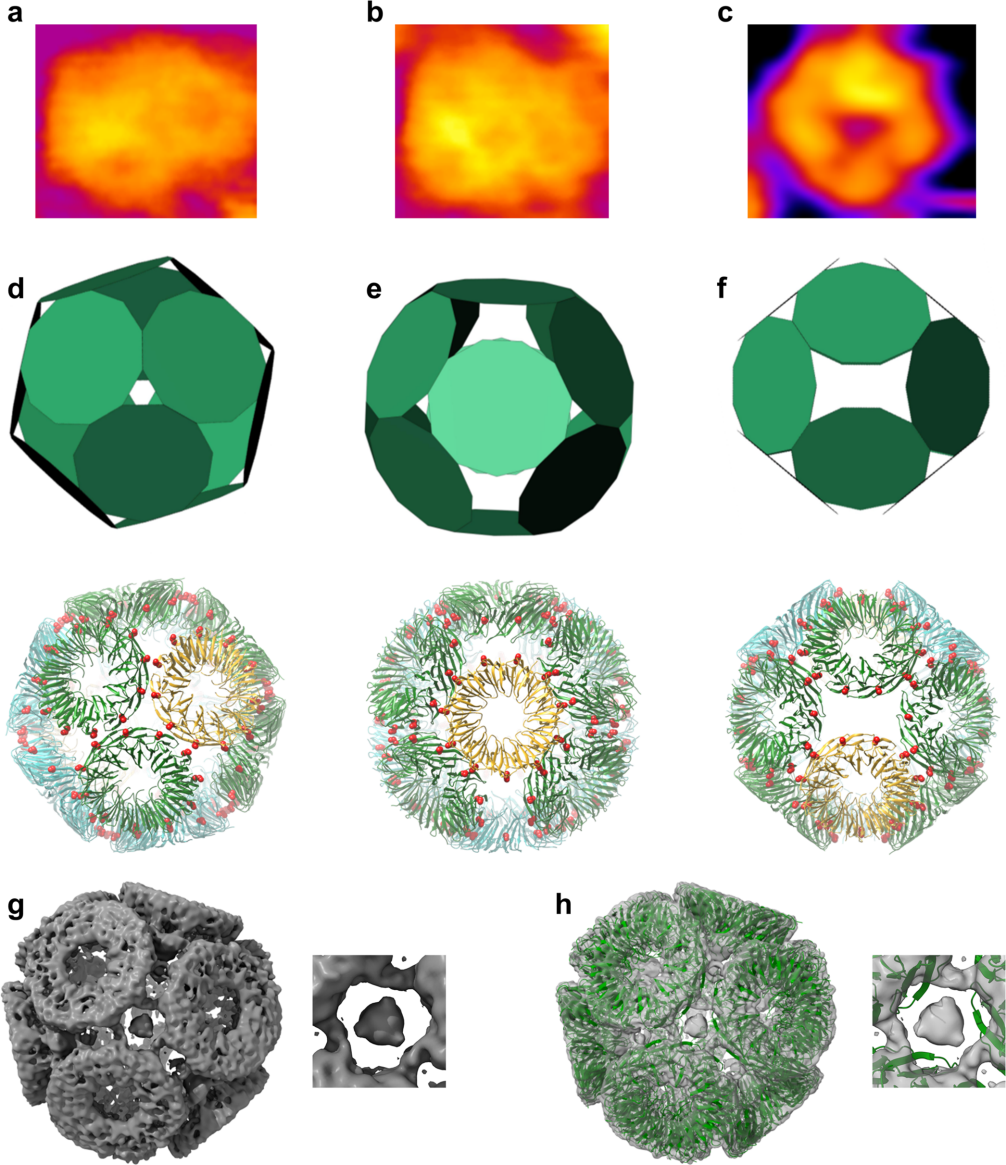
Structure of TRAP-SC^GNP^. (a–c) Three most commonly observable topographies of TRAP-SC^GNP^ under the HS-AFM. (d–f) Three views of a predicted possible structure of the small cage shown as abstract convex shapes with flat hendecagonal faces (top) and as the equivalent structures built using the known structure of the TRAP 11-mer (PDB: 4 V4F)^37^ (bottom) centered at (d) the 3-fold hole, (e) the TRAP ring, and (f) the 2-fold hole. Residue C35 in TRAP rings are depicted as red spheres. For a summary of the features of the predicted structures, see Table S2. (g,h) Cryo-EM structure of TRAP-SC^GNP^ (EMD-12526). (g) Overall cryo-EM map of TRAP-SC^GNP^ at SD = 3.5 contouring level and (h) pseudoatomic model built inside the obtained electron density. Inserts to the right show close-up view of additional electron density in the center of the 3-fold axes. Bridging densities lying between neighboring TRAP rings are also visible.

The collected data allowed us to estimate the total number of TRAP rings in the structure and build a speculative model of the arrangement of TRAP rings in the TRAP-SC^GNP^ cage supported by the AFM results (Figure S6, Figure 3a–f). Such a model could then be compared against experimentally obtained high-resolution structural data (see next section) allowing us to assess the usefulness of the mathematical model in predicting real arrangements of rings. To build this model, we assumed a maximum of 15 rings per cage. We then harnessed the algorithm used in Malay et al.^14^ and assumed the same type of connections between the rings as in TRAP-LC^GNP^. We also expect every ring to have the same connections as every other ring which excludes 13 to 15 ring situations and leaves us with a maximum of 12 rings (see Supporting Information, Experimental Methods for details). The only model with 12 rings that matches the anticipated size and angles between pairs of rings is the one presented here. The diameter is approximately 16.5 nm and the angle between adjacent faces is 119° and 101° between two faces separated by a hole. Its construction is based on Archimedean cuboctahedron, but the final model lacks the 4-fold axis; it only has approximately three 2-fold and four 3-fold axes of symmetry. The predicted model shows small levels of deviations from regularity. These can be expressed as relative deformations, termed rdl and rda, defined as the largest absolute value of the difference between the edge lengths and the average edge length, divided by the average value (rdl); similarly for the angles (rda) for TRAP-SC^GNP^, we obtain rdl = 0% and rda = 1.83%.

## Structural Determination using Cryo-EM

Given the predictions that TRAP-SC^GNP^ would form a unique arrangement of hendecamer rings, we determined the cryo-EM structure of TRAP-SC^GNP^ (EMD-12526, Figure 3g,h, Figures S7–9). The density maps clearly show that the cage consists of 12 TRAP rings. These are arranged to an approximation as the equivalent of one TRAP ring on each of the 12 faces of a dodecahedron. Each ring is surrounded by five others, as in TRAP-LC^GNP^; however, in the latter this arrangement results in each TRAP ring forming one edge of six large “square” holes, approximately 4 nm across, in the cage. The predicted structures of TRAP-SC^GNP^ also contain large bowtie-shaped holes but these are absent from the experimentally determined structure due to the presence of additional electron density (possibly due to gold) that fills up the holes, and the fact that the protein is distorted (Figures S10–11). The final arrangement of the rings is somewhat a compromise between the overall symmetry and the level of distortion when a high level of (undistorted) symmetry is impossible to achieve and illustrates how protein flexibility and variability in “cross-linker” lengths can accommodate significant deviation from ideality.

**Figure 4:**
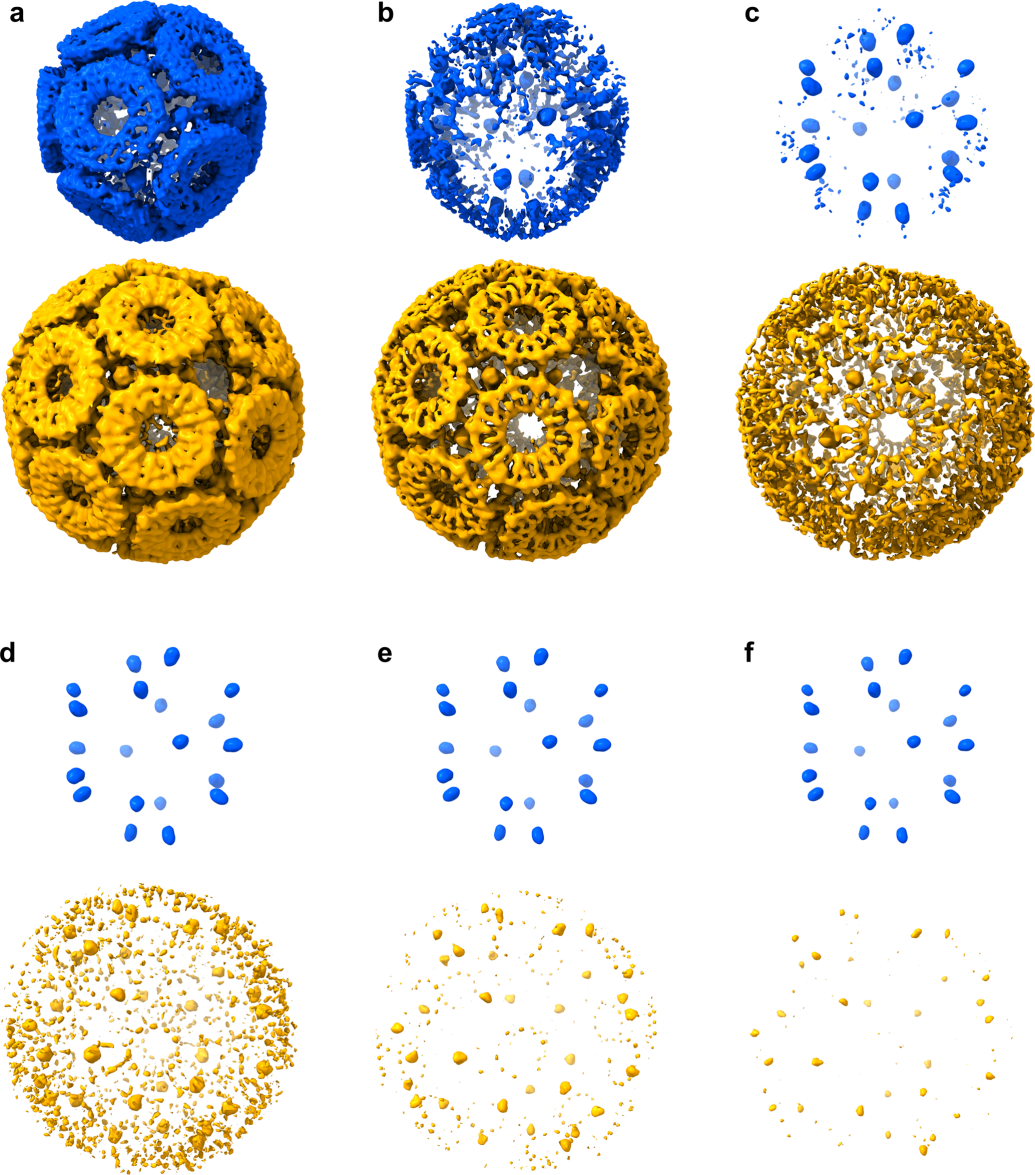
TRAP-SC^GNP^ is a scaffold for a regular 3D arrangement of GNPs. (a–f) Cryo-EM maps of TRAP-SC^GNP^ (upper panels, blue, panel a showing same data as in Figure 3 panel g) and TRAP-LC^GNP^ (EMD-6966)^14^ (lower panels, gold) at different SD countouring levels: (a) SD = 3.5, (b) SD = 4.5, (c) SD = 5.5, (d) SD = 6.5, (e) SD = 7.5, and (f) SD = 8.5. Panel f clearly shows densities of GNPs with a stark difference in density between the two cage types.

Notably, we clearly observe the presence of GNPs in the cage structure. Specifically, we see 20 GNPs, each one between three rings at positions which resemble the 20 vertices of a dodecahedron (meaning that five GNPs surround each TRAP ring, Figure 4). While some evidence of the presence of GNPs has been noted in TRAP-LC^GNP^, the occupancy in these new structures is much higher as clearly shown in Cryo-EM density contoured at different SD levels. In the case of TRAP-LC^GNP^ (EMD-6966),^14^ the protein part of the density disappears at about SD = 7.5, while for TRAP-SC^GNP^ setting the SD threshold at 5.5 is sufficient to hide the protein density almost completely (Figure 4).

Closer inspection of the cryo-EM structure revealed unexpected details of the connections between TRAP rings. First, the distance between opposing sulphurs of cysteine side chains of TRAP-SC^GNP^ was found to be 10–20 Å, considerably greater than the 5 Å seen for TRAP-LC^GNP^ and too great to be the same form of S–Au–S linear coordinate bond. Rather, it suggests two or more bridging gold ion “staples”.^30^ The cysteines bridged by gold staples account for connections between 9 of the 11 cysteines of each ring. We were able to distinguish three distinct linkages which we named Type I–III: Type I is very similar to the links previously described in Malay et al.^14^ being direct Cys-gold–Cys bridges but likely consisting of 2 or 3 gold ions instead of one (Figure S11). These connections can be modeled as edge-to-edge interactions between hendecagons (Figure S11b). Type II connections, not seen in TRAP-LC^GNP^, are the equivalent of vertex-to-vertex connections, located between two GNPs (Figure S11c), and tend to be slightly longer (∼12 Å) than Type I (10–11 Å). Type III bonds consist of pairs of bridged cysteines where the bridged sulphurs are not directly opposite but are on a diagonal, giving the bonding pattern a zipperlike appearance (Figure S11d). Here, distances between Cys residues range from ∼15 to ∼20 Å.

Protein and other biomolecular structures have previously been shown to be able to interact with source of gold ions allowing, for example, the reductive formation of gold nanoclusters within the lumen of a ferritin cage,^31^ while others have shown to externally anchor GNPs,^32^ (including the cagelike cowpea mosaic virus VLP^33^). However, the presence of an embedded, ordered arrangement of GNPs in the structure observed in our case was not expected and as far as we are aware has never before been observed, although the large TRAP cage structure had suggested that some could be weakly bound.^14^ In fact, local resolution calculations revealed that the most rigid part of the assembled cages are the GNPs, while the protein part is more flexible, thus local resolution of the TRAP rings is much lower than it appears from gold standard FSC plots (Figure S7). The main reason for this may be the fact that GNPs are very electron dense and internally rigid.

With current resolution limitations, we are unable to directly discern discrete gold ions bridging the sulphurs of cysteine side chains directly. However, assuming that the average S–Au distance is 2.4 Å and the average Au–Au distance is 2.9 Å and considering previously published theoretical calculations (Figure S11a), we can conclude that the gold bridges have up to nine gold atoms (Figure S11b–d). These are arranged in varying conformations with longer or shorter linear spans or in a zippered pattern. The presence of bridges consisting of multiple gold ions gives the capacity from additional bonds beyond those between cysteine thiols. Indeed, densities are visible in the cryo-EM maps which connect the surface of the embedded GNPs to the expected location of the bridging golds (Figure S10), suggesting that such bonds are responsible for holding the GNPs in place.

Differences between the mathematical model and determined structure may be explained by the fact that in the mathematical modeling process we use planar hendecagonal plates that can only change the position of vertices in a way that planarity is sustained. We do not model the protein ring flexibility, hence the mathematical structure does not take into account any “twisting” of the rings. Furthermore, the mathematical model assumes that every ring is connected to its neighbors in an identical way (and using exactly two adjacent points of contact). Expanding the model to allow a prediction of a different neighborhood for several groups of rings or only one point of contact between the rings, then predictions of increased accuracy are likely.

Overall, we have shown that TRAP-SC^GNP^ is an unusual artificial protein cage. Its geometrical arrangement represents an interesting solution posed by the incompatibility of a hendecagon with formation of a regular-faced convex polyhedron that goes beyond the minor distortion required to form the “snub cube” arrangement of 24 rings.^14^ The multiatom gold staples in the structure appear to endow the cage with high stability. However, the cages readily disassemble in the presence of DTT, presumably through a gold etching reaction. Furthermore, the small cage has a considerable spring constant, which is mainly due to its relatively thick shell. The overall material properties (Young’s modulus) of the cage are rather on the soft side due to the relation between the spring constant, Young’s modulus, and shell dimensions.^29^

These results raise interesting questions for future research. We have shown that while TRAP-cages are highly stable they are also polymorphic; they can be switched between “large” and “small” versions by simple manipulation of the concentration of gold added to assembly reactions while keeping the protein component unchanged. Similar polymorphism caused by simple changes in buffer have been reported for some viruses.^34,35^ This is an interesting complement to earlier work which demonstrated that assembling VLPs around a GNP core could control the VLP size depending on the size of GNP used.^36^ In our work, much smaller gold particles/atoms rather than GNPs interact at protein–protein interfaces rather than the central lumen. An understanding of the detailed process of how this is achieved will require more in depth kinetic studies. Furthermore, the physical and chemical properties of the ordered GNP lattice are yet to be investigated and are particularly intriguing given the known catalytic activity of GNPs in this size range.^32^

## Supporting Information Available

Details of experimental methods, TRAP-SC^GNP^ full characterization (purification, stability tests, TEM, AFM, DLS, cryo-EM), comparison between large and small cage, and GNPs incorporation in the TRAP-SC structure (PDF)
Stability of TRAP-SC^GNP^ monitored by HS-AFM (AVI)
Disassembly of TRAP-SC^GNP^ in the presence of 3 mM DTT monitored by HS-AFM (AVI)

## Supplementary Material

nl1c04222_si_001.pdf

nl1c04222_si_002.avi

nl1c04222_si_003.avi

## Notes

The cryo-EM density map has been deposited in the Electron Microscopy Data Bank under accession code EMD-12526 (TRAP-SC^GNP^).

## ABBREVIATIONS

AFM: Atomic Force Microscopy
cryo-EM: Cryogenic Electron Microscopy
Cys: Cysteine
DLS: Dynamic Light Scattering
DTT: 1,4-Dithiothreitol
GNPs: Gold Nanoparticles
GSH: l-Glutathione reduced
GSSG: Glutathione oxidized
HS-AFM: High-Speed AFM
RALS/LALS: Right-Angle Light Scattering/Low-Angle Light Scattering
SDS: Sodium Dodecyl Sulfate
SEC: Size Exclusion Chromatography
TCEP: tris(2-carboxyethyl)phosphine
TEM: Transmission Electron Microscopy
TPPMS: Triphenylphosphine Mono-Sulfonated (3-(diphenylphosphino)benzenesulfonic acid sodium salt)
TRAP: trp RNA-binding Attenuation Protein
TRAPCS: TRAP(K35C/R64S) mutant
TRAP-LC^GNP^: TRAP-Large Cage formed by incubation of TRAPCS with GNPs
TRAP-SC^GNP^: TRAP-Small Cage formed by incubation of TRAPCS with GNPs
VLP: Virus-Like Particle

## References

[ref1] Pulsipher K. W.; Dmochowski I. J. Ferritin: Versatile Host, Nanoreactor, and Delivery Agent. Isr. J. Chem. 2016, 56 (9–10), 660–670. 10.1002/ijch.201600017.

[ref2] Chung Y. H.; Cai H.; Steinmetz N. F. Viral Nanoparticles for Drug Delivery, Imaging, Immunotherapy, and Theranostic Applications. Adv. Drug Delivery Rev. 2020, 156, 214–235. 10.1016/j.addr.2020.06.024. PMC7320870 32603813

[ref3] Antson A. A.; Dodson E. J.; Dodson G.; Greaves R. B.; Chen X. P.; Gollnick P. Structure of the Trp RNA-Binding Attenuation Protein, TRAP, Bound to RNA. Nature 1999, 401 (6750), 235–242. 10.1038/45730. 10499579

[ref4] Antson A. A.; Otridge J.; Brzozowski A. M.; Dodson E. J.; Dodson G. G.; Wilson K. S.; Smith T. M.; Yang M.; Kurecki T.; Gollnick P. The Structure of Trp RNA-Binding Attenuation Protein. Nature 1995, 374 (6524), 693–700. 10.1038/374693a0. 7715723

[ref5] Gollnick P.; Babitzke P.; Antson A.; Yanofsky C. Complexity in Regulation of Tryptophan Biosynthesis in Bacillus Subtilis. Annu. Rev. Genet. 2005, 39, 47–68. 10.1146/annurev.genet.39.073003.093745. 16285852

[ref6] Malay A. D.; Watanabe M.; Heddle J. G.; Tame J. R. H. Crystal Structure of Unliganded TRAP: Implications for Dynamic Allostery. Biochem. J. 2011, 434 (3), 427–434. 10.1042/BJ20101813. 21175426 PMC3048579

[ref7] Watanabe M.; Heddle J. G.; Kikuchi K.; Unzai S.; Akashi S.; Park S.; Tame J. R. H. The Nature of the TRAP – Anti-TRAP Complex. Proc. Natl. Acad. Sci. U.S.A. 2009, 106 (7), 2176–2181. 10.1073/pnas.0801032106. 19164760 PMC2650128

[ref8] Heddle J. G.; Okajima T.; Scott D. J.; Akashi S.; Park S. Y.; Tame J. R. H. Dynamic Allostery in the Ring Protein TRAP. J. Mol. Biol. 2007, 371 (1), 154–167. 10.1016/j.jmb.2007.05.013. 17559872

[ref9] Heddle J. G.; Fujiwara I.; Yamadaki H.; Yoshii S.; Nishio K.; Addy C.; Yamashita I.; Tame J. R. H. Using the Ring-Shaped Protein TRAP to Capture and Confine Gold Nanodots on a Surface. Small 2007, 3 (11), 1950–1956. 10.1002/smll.200700400. 17935079

[ref10] Miranda F. F.; Iwasaki K.; Akashi S.; Sumitomo K.; Kobayashi M.; Yamashita I.; Tame J. R. H.; Heddle J. G. A Self-Assembled Protein Nanotube with High Aspect Ratio. Small 2009, 5 (18), 2077–2084. 10.1002/smll.200900667. 19562822

[ref11] Nagano S.; Banwell E. F.; Iwasaki K.; Michalak M.; Pałka R.; Zhang K. Y. J.; Voet A. R. D.; Heddle J. G. Understanding the Assembly of an Artificial Protein Nanotube. Adv. Mater. Interfaces 2016, 3 (24), 1600846 10.1002/admi.201600846.

[ref12] Imamura M.; Uchihashi T.; Ando T.; Leifert A.; Simon U.; Malay A. D.; Heddle J. G. Probing Structural Dynamics of an Artificial Protein Cage Using High-Speed Atomic Force Microscopy. Nano Lett. 2015, 15 (2), 1331–1335. 10.1021/nl5045617. 25559993

[ref13] Malay A. D.; Heddle J. G.; Tomita S.; Iwasaki K.; Miyazaki N.; Sumitomo K.; Yanagi H.; Yamashita I.; Uraoka Y. Gold Nanoparticle-Induced Formation of Artificial Protein Capsids. Nano Lett. 2012, 12 (4), 2056–2059. 10.1021/nl3002155. 22414047

[ref14] Malay A. D.; Miyazaki N.; Biela A.; Chakraborti S.; Majsterkiewicz K.; Stupka I.; Kaplan C. S.; Kowalczyk A.; Piette B. M. A. G.; Hochberg G. K. A.; Wu D.; Wrobel T. P.; Fineberg A.; Kushwah M. S.; Kelemen M.; Vavpetič P.; Pelicon P.; Kukura P.; Benesch J. L. P.; Iwasaki K.; Heddle J. G. An Ultra-Stable Gold-Coordinated Protein Cage Displaying Reversible Assembly. Nature 2019, 569 (7756), 438–442. 10.1038/s41586-019-1185-4. 31068697

[ref15] Piette B. M. A. G.; Kowalczyk A.; Heddle J. G. Characterisation of Near-Miss Connectivity-Invariant Homogeneous Convex Polyhedral Cages. Proc. R. Soc. A Accepted for publication. 10.1098/rspa.2021.0679 PMC8984814 35450023

[ref16] Liz-Marzán L. M. Tailoring Surface Plasmons through the Morphology and Assembly of Metal Nanoparticles. Langmuir 2006, 22 (1), 32–41. 10.1021/la0513353. 16378396

[ref17] Georgiev P.; Simeonova S.; Tsekov R.; Balashev K. Dependence of Plasmon Spectra of Small Gold Nanoparticles from Their Size: An Atomic Force Microscopy Experimental Approach. Plasmonics 2020, 15 (2), 371–377. 10.1007/s11468-019-01034-4.

[ref18] Prasad B. L. V.; Sorensen C. M.; Klabunde K. J. Gold Nanoparticle Superlattices. Chem. Soc. Rev. 2008, 37 (9), 1871–1883. 10.1039/b712175j. 18762836

[ref19] Zhang H.; Cadusch J.; Kinnear C.; James T.; Roberts A.; Mulvaney P. Direct Assembly of Large Area Nanoparticle Arrays. ACS Nano 2018, 12 (8), 7529–7537. 10.1021/acsnano.8b02932. 30004661

[ref20] Valbuena A.; Maity S.; Mateu M. G.; Roos W. H. Visualization of Single Molecules Building a Viral Capsid Protein Lattice through Stochastic Pathways. ACS Nano 2020, 14 (7), 8724–8734. 10.1021/acsnano.0c03207. 32633498 PMC7392527

[ref21] Buzón P.; Maity S.; Roos W. H. Physical Virology: From Virus Self-Assembly to Particle Mechanics. In Wiley Interdisciplinary Reviews: Nanomedicine and Nanobiotechnology; Wiley-Blackwell, 2020; p e1613. 10.1002/wnan.1613 PMC7317356 31960585

[ref22] Snijder J.; Uetrecht C.; Rose R. J.; Sanchez-Eugenia R.; Marti G. A.; Agirre J.; Guérin D. M. A.; Wuite G. J. L.; Heck A. J. R.; Roos W. H. Probing the Biophysical Interplay between a Viral Genome and Its Capsid. Nat. Chem. 2013, 5 (6), 502–509. 10.1038/nchem.1627. 23695632

[ref23] Hernando-Pérez M.; Miranda R.; Aznar M.; Carrascosa J. L.; Schaap I. A. T.; Reguera D.; De Pablo P. J. Direct Measurement of Phage Phi29 Stiffness Provides Evidence of Internal Pressure. Small 2012, 8 (15), 2366–2370. 10.1002/smll.201200664. 22648860

[ref24] Denning D.; Bennett S.; Mullen T.; Moyer C.; Vorselen D.; Wuite G. J. L.; Nemerow G.; Roos W. H. Maturation of Adenovirus Primes the Protein Nano-Shell for Successful Endosomal Escape. Nanoscale 2019, 11 (9), 4015 10.1039/C8NR10182E. 30768112

[ref25] de Pablo P. J.; Mateu M. G. Mechanical Properties of Viruses. Subcell. Biochem. 2013, 68, 519–551. 10.1007/978-94-007-6552-8_18. 23737064

[ref26] Roos W. H.; Gibbons M. M.; Arkhipov A.; Uetrecht C.; Watts N. R.; Wingfield P. T.; Steven A. C.; Heck A. J. R.; Schulten K.; Klug W. S.; Wuite G. J. L. Squeezing Protein Shells: How Continuum Elastic Models, Molecular Dynamics Simulations, and Experiments Coalesce at the Nanoscale. Biophys. J. 2010, 99 (4), 1175–1181. 10.1016/j.bpj.2010.05.033. 20713001 PMC2920642

[ref27] Uetrecht C.; Versluis C.; Watts N. R.; Roos W. H.; Wuite G. J. L.; Wingfield P. T.; Steven A. C.; Heck A. J. R. High-Resolution Mass Spectrometry of Viral Assemblies: Molecular Composition and Stability of Dimorphic Hepatitis B Virus Capsids. Proc. Natl. Acad. Sci. U. S. A. 2008, 105 (27), 9216–9220. 10.1073/pnas.0800406105. 18587050 PMC2453694

[ref28] Ivanovska I. L.; De Pablo P. J.; Ibarra B.; Sgalari G.; MacKintosh F. C.; Carrascosa J. L.; Schmidt C. F.; Wuite G. J. L. Bacteriophage Capsids: Tough Nanoshells with Complex Elastic Properties. Proc. Natl. Acad. Sci. U. S. A. 2004, 101 (20), 7600–7605. 10.1073/pnas.0308198101. 15133147 PMC419652

[ref29] Roos W. H.; Bruinsma R.; Wuite G. J. L. Physical Virology. Nat. Phys. 2010, 6 (10), 733–743. 10.1038/nphys1797.

[ref30] Zhao Y.; Zhou F.; Zhou H.; Su H. The Structural and Bonding Evolution in Cysteine-Gold Cluster Complexes. Phys. Chem. Chem. Phys. 2013, 15 (5), 1690–1698. 10.1039/C2CP42830J. 23247849

[ref31] Maity B.; Abe S.; Ueno T. Observation of Gold Sub-Nanocluster Nucleation within a Crystalline Protein Cage. Nat. Commun. 2017, 8, 14820 10.1038/ncomms14820. 28300064 PMC5357307

[ref32] Heddle J. G. Gold Nanoparticle-Biological Molecule Interactions and Catalysis. Catalysts; Multidisciplinary Digital Publishing Institute, 2013; pp 683–708.

[ref33] Blum A. S.; Soto C. M.; Wilson C. D.; Cole J. D.; Kim M.; Gnade B.; Chatterji A.; Ochoa W. F.; Lin T.; Johnson J. E.; Ratna B. R. Cowpea Mosaic Virus as a Scaffold for 3-D Patterning of Gold Nanoparticles. Nano Lett. 2004, 4 (5), 867–870. 10.1021/nl0497474.

[ref34] Salunke D. M.; Caspar D. L.; Garcea R. L. Polymorphism in the Assembly of Polyomavirus Capsid Protein VP1. Biophys. J. 1989, 56 (5), 887–900. 10.1016/S0006-3495(89)82735-3. 2557933 PMC1280588

[ref35] Kanesashi S. N.; Ishizu K. I.; Kawano M. A.; Han S. I.; Tomita S.; Watanabe H.; Kataoka K.; Handa H. Simian Virus 40 VP1 Capsid Protein Forms Polymorphic Assemblies in Vitro. J. Gen. Virol. 2003, 84 (7), 1899–1905. 10.1099/vir.0.19067-0. 12810885

[ref36] Sun J.; DuFort C.; Daniel M. C.; Murali A.; Chen C.; Gopinath K.; Stein B.; De M.; Rotello V. M.; Holzenburg A.; Kao C. C.; Dragnea B. Core-Controlled Polymorphism in Virus-like Particles. Proc. Natl. Acad. Sci. U. S. A. 2007, 104 (4), 1354–1359. 10.1073/pnas.0610542104. 17227841 PMC1783121

[ref37] Hopcroft N. H.; Manfredo A.; Wendt A. L.; Brzozowski A. M.; Gollnick P.; Antson A. A. The Interaction of RNA with TRAP: The Role of Triplet Repeats and Separating Spacer Nucleotides. J. Mol. Biol. 2004, 338 (1), 43–53. 10.1016/j.jmb.2004.02.038. 15050822

