# Supplementary material for: Artificial Protein Cage with Unusual Geometry and Regularly Embedded Gold Nanoparticles
Source: Nano Lett. 2022 Mar 7;22(8):3187–95. doi: 10.1021/acs.nanolett.1c04222 (PMC9052746; doi:10.1021/acs.nanolett.1c04222)
Supplement: Supplementary file 1 — nl1c04222_si_001.pdf [file nl1c04222_si_001.pdf]

Supporting Information

An artificial protein cage with unusual geometry and regularly embedded gold nanoparticles

Karolina Majsterkiewicz[1,2,], Artur P. Biela[1,3], Sourav Maity[4], Mohit Sharma[1,2], Bernard M. A. G. Piette[5], Agnieszka Kowalczyk[1,6], Szymon Gaweł[1], Soumyananda Chakraborti[1,‡], Wouter H. Roos[4], Jonathan G. Heddle[1]*

[1]Małopolska Centre of Biotechnology, Jagiellonian University, Kraków 30-387, Poland

[2]Postgraduate School of Molecular Medicine, ul. Żwirki i Wigury 61, Warsaw, 02-091, Poland

[3]Institute of Zoology and Biomedical Research, Department of Cell Biology and Imaging, Jagiellonian University, Kraków 30-387, Poland

[4]Moleculaire Biofysica, Zernike Instituut, Rijksuniversiteit Groningen, Groningen 9747 AG, Netherlands

[5]Department for Mathematical Sciences, Durham University, Durham DH1 3LE, United Kingdom

[6]Faculty of Mathematics and Computer Science, Jagiellonian University, Kraków 30-348, Poland

Corresponding Author

[*]email: jonathan.heddle@uj.edu.pl

Present Addresses

[‡]National Institute of Malaria Research, New Delhi, India

MATERIALS AND METHODS

Protein expression and purification. TRAP(K35C/R64S) protein was expressed and purified as described previously.[1] Protein concentration was determined by measuring absorbance at 280 nm. Exemplary chromatograms and denaturing gels are presented on **Figure S12**.

Gold nanoparticles. The gold nanoparticles (GNPs) used were diphenyl(*m*-sulfonatophenyl)phosphine-gold nanoclusters with a 1–3 nm core diameter (MDL number MFCD17018839) from STREM Chemicals UK and reconstituted in water to 5 mM stock concentration.

Cage assembly with GNPs. Formation of small TRAP-cage (TRAP-SC^{GNP}) was carried out by mixing purified TRAP(K35C/R64S) (final concentration 0.1 mM of monomeric subunits) with excess GNPs in a ratio between 1:2 – 1:4 in 50 mM Tris-HCl, pH 7.9, 0.15 M NaCl buffer (cage buffer). Reactions were typically incubated for 3 days at room temperature (RT). Formation of TRAP-cage was confirmed using native PAGE. Any precipitated material (aggregated protein, present in older samples or due to excess GNPs) was removed by centrifugation at 12,045 g for 5 min. Excess GNPs were removed by washing the TRAP-cage sample on Amicon Ultra 30 kDa MWCO centrifugal filter units (Millipore) with cage buffer 4 times, then concentrating and passing through a 0.1 μm PVDF centrifugal filter (Millipore) followed by size-exclusion chromatography (SEC) on a Superose 6 Increase 10/300 GL column (GE Healthcare). Small cage typically was eluted with a peak maximum at around 13.0 – 14.0 ml. Fractions containing pure small cage were pooled and protein concentration was assessed by comparison of band intensities on denaturing SDS gels for cage and unreacted TRAP(K35C/R64S) protein. Comparisons were made using ImageJ software (**Figure S13**).

Formation of large TRAP-cage (TRAP-LC^{GNP}) was carried out in similar fashion, but the reaction consisted of equimolar amounts of TRAP(K35C/R64S) (final concentration 1 mM of monomeric subunits) and GNPs in cage buffer. SEC purification typically resulted in large cage eluting with a peak maximum at around 12.0 – 13.0 ml.

Cage stability. Final concentration of protein for stability tests was around 0.1 mg/ml. Purified cage stock was prepared in cage buffer for all the tests and mixed with tested agents, ensuring that the volume of the cage stock constituted less than 10% of the total volume. After each test was finished, samples were centrifuged and supernatant containing ~1 µg of protein was mixed with 4X native PAGE sample buffer. Results were monitored by dark blue native PAGE. Supernatant of selected samples was also analysed by TEM. Each test was repeated at least once with similar results.

For thermal tests, each sample was incubated for 10 min. in temperatures between 45-90°C in cage buffer. pH stability tests were performed by diluting cage stock with suitable buffers and incubating overnight at room temperature. Buffers used: 50 mM Gly-HCl, pH 2 or 3; 50 mM sodium acetate, pH 4; 50 mM potassium phosphate, pH 6 or 12; 50 mM Gly-NaOH, pH 10 or 11; 50 mM KCl-NaOH, pH 13.

Chaotropic agents' stocks (SDS, urea, Gdn-HCl) were prepared in 50 mM Tris-HCl, pH 7.9. Tested agents were diluted to suitable concentrations with the same buffer, mixed with cage stock and incubated overnight at room temperature.

Cage disassembly was tested with oxidised (GSSG) and reduced (GSH) forms of glutathione, DTT, TCEP, L-Cys and 3-(diphenylphosphino)benzenesulfonic acid sodium salt (TPPMS). Stocks of tested chemicals were prepared in water or cage buffer, pH adjusted if needed, then diluted to

suitable concentrations with cage buffer, mixed with cage stock and incubated overnight at room temperature.

Dark blue native PAGE. Electrophoresis was performed either on manually cast gels or commercial gels. When commercial gels were used, samples were mixed with 4X sample buffer (200 mM Bis-Tris-HCl, pH 7.2; 40% [w/v] glycerol; 0.06% [v/v] bromophenol blue) and run on the gels (Life Technologies; NativePAGE™ Novex® 3–12% Bis-Tris Gels) in running buffer (Life Technologies; NativePAGE™ Running Buffer (20X)), where the cathode buffer was supplemented with 0.02% [w/v] Coomassie Brilliant Blue G-250. When manually cast gels were used, samples were mixed with 4X sample buffer (125 mM Tris-HCl, pH 8.9; 40% [w/v] glycerol; 0.04% [v/v] bromophenol blue) and run on manually cast gels (final concentrations: 12, 9, 5, 4, 3% layers of 37.5:1 acrylamide:bisacrylamide; 375 mM Tris-HCl, pH 8.8) in running buffer (final concentrations: 25 mM Tris; 192 mM Gly; pH not adjusted), where cathode buffer was supplemented with 0.02% [w/v] Coomassie Brilliant Blue G-250. In both cases gels were fixed in 40% methanol, 10 % acetic acid (microwaved for 45 s, 700 W, then gently shaken for at least 15 min.) and destained in 8% acetic acid (microwaved for 45 s, 700 W, then gently shaken until desired background was achieved). Molecular weights of migrated bands were estimated using NativeMark unstained protein standard (Life Technologies; NativeMark™ Unstained Protein Standard).

Denaturing SDS- PAGE. Samples were mixed with 4X sample buffer (200 mM Tris-HCl, pH 6.8; 40% [w/v] glycerol; 8% SDS; 40 mM β-mercaptoethanol; 0.4% [v/v] bromophenol blue), heated at 95°C for 10 min. and run on manually cast gels (final concentrations: 16, 10, 4% layers of 37.5:1 acrylamide:bisacrylamide; 1 M Tris-HCl, pH 8.45; 0.1% [w/v] SDS) in running buffer (final concentrations for cathode buffer: 0.1 M Tris; 0.1 M Tricine; 0.1% [w/v] SDS; pH not

adjusted; final concentrations for anode buffer: 0.5 M Tris-HCl, pH 8.9). To ensure complete denaturation of protein when estimating cage concentration, 5 μL of sample was mixed with 5 μL of 4X sample buffer supplemented with 0.2 M DTT and 10 μL of saturated urea, then heated in 95°C for 20 min. Gels were stained in InstantBlue™ (Expedeon). Molecular weights of migrated bands were estimated using prestained protein ladder (Thermo Scientific; PageRuler™ Plus Prestained Protein Ladder).

***Dynamic light scattering (DLS*)**. Dynamic light scattering was carried out using a Zetasizer Nano ZS (Malvern). Small cage samples in cage buffer were centrifuged for 5 min. at 12,045 g prior to measurements. 50 μl aliquots were placed in disposable cuvettes (ZEN 0040) and preincubated for 120 s at 25°C. The samples were measured with standard settings for 50 mM Tris-HCl, 0.15 M NaCl buffer. Results were given as volume distributions.

***Transmission electron microscopy (TEM*)**. TEM grids (Formvar/Carbon film-Cu 300 mesh, FC300Cu100, EMResolutions) were glow-discharged in grid mode for 70 s, 8 mA (Leica EM ACE200). Samples for TEM imaging were typically diluted to a final protein concentration of 0.10 mg/ml, 3 μl applied onto prepared grids, negatively stained with 2 μl freshly prepared 3% phosphotungstic acid, pH 7-8, and visualized using a JEOL JEM-1230 80 kV instrument. Cage size measurements and statistics were carried out using ImageJ software.

***Atomic force microscopy (AFM) based nanoindentation*.** AFM based nanoindentation experiments[2,3] were performed using a NanoWizard Ultra-speed AFM (JPK, Berlin). Qp-BioAC (NanoAndMore) cantilevers were used with a nominal spring constant of 0.1 N/m. TRAP-cage samples (purified) were first diluted 50-fold from the stock (see above: Cage assembly with GNPs) in recording buffer (25 mM Tris, pH 7.4), and finally deposited onto a Highly Ordered Pyrolytic

Graphite (HOPG) surface for 15-30 minutes. Samples were visualized under aqueous conditions and at room temperature by QI mode. The nanoindentation experiments were done in closed loop contact mode with a tip speed of 500 nm/s, and an indentation force of 1 nN. Each indented particle was imaged before and after indentation.

The acquired images and force-distance (F-D) curves were processed using JPK data processing software. The determination of the particle spring constant was performed using IgorPro software by following the protocol as described in reference.[2] The F-D curves extracted from indenting the particle shows linear behaviour. Therefore, we used Hooke's law considering two springs in series (particle and cantilever) in order to extract the particle spring constant. Reported errors are standard deviation. Using the averaged dimensional parameters (diameter and thickness) of the particles (both TRAP-SC^{GNP} and TRAP-LC^{GNP}) and the particle spring constant, the Young's modulus was determined by thin cell theory.[2] For this calculation the thickness of the shell was taken and as radius, the outer radius minus half the thickness.

High Speed-AFM **(HS-AFM).** The major challenge in the structural analysis of TRAP-cages using AFM is its structural diversity within a very small particle surface area (particle diameter of 15-22 nm). Using HS-AFM images at high throughput were recorded, with high signal to noise ratio, and at minimal imaging force (<100 pN). HS-AFM (RIBM, Japan) was used in amplitude modulation tapping mode. USC-F1.2-k0.15 cantilevers (Nanoworld) with a nominal spring constant of 0.15 N/m were used.[4,5] The acquired images were processed using Kodec (Kanazawa University, Japan), and ImageJ software. Unless otherwise mentioned, the images were processed minimally (i.e. baseline correction, contrast check etc.).

The estimation of dihedral angle between the rings was calculated using published procedures.[6] In brief, in reference to **Figure S5**, the calculation steps are as follows:

Step 1 contains the measurements of corrected distances (in x, y and z direction) between two consecutive points. The X, Y, and Z position of the observable ring center (on a cage) was recorded from the images, and distances between two points were calculated ($x_{measured}$ and $y_{measured}$). The corresponding measured distances were corrected for tip convolution as follows:

$$x_{corrected} = x_{measured} \frac{1}{n} \sum \frac{2 \cdot r(z)_{measured}}{r(x)_{i(measured)} + r(y)_{i(measured)}} \quad (1)$$

$$y_{corrected} = y_{measured} \frac{1}{n} \sum \frac{2 \cdot r(z)_{measured}}{r(x)_{i(measured)} + r(y)_{i(measured)}} \quad (2)$$

Where n is the number of measurements in X and Y direction for each particle. $r(z)_{measured}$, $r(x)_{i(measured)}$, and $r(y)_{i(measured)}$ are the measurements of the height, x diameter, and y diameter of the corresponding particles, respectively.

In *step 2*, the calculation of the actual distances (D) between the two ring centers are estimated as follows:

$$D_{measured\&corrected} = \sqrt{(x_{corrected}^2 + y_{corrected}^2 + z_{measured}^2)} \quad (3)$$

In *step 3*, the dihedral angle between the ring is calculated as follows:

$$Dihedral\ angle\ between\ two\ rings = \cos^{-1}\left(\frac{2 \cdot ring\ diameter^2 - D_{measured\&corrected}^2}{2 \cdot ring\ diameter^2}\right) \quad (4)$$

Size Exclusion Chromatography with Right-Angle (RALS)/Low-Angle (LALS) Light Scattering and Refractive Index (RI) detection. Molecular mass analysis was performed by subjecting purified TRAP-SC^{GNP} sample in cage buffer containing 150 μg of protein (0.75 mg/ml)

to SEC on a Superose 6 Increase 10/300 GL column (GE Healthcare) in RT with RALS/LALS and RI detection (OMNISEC REVEAL, Malvern Panalytical Ltd) and data was processed with Omnisec software (v11.10.7248.3). Parameters used for the run were as follows: 0.4 ml/min. flow rate, total run time 70 min., sample type – protein in aqueous (dn/dc = 0.185; dA/dC = 0; Second virial coefficient (A2) = 0), solvent – Tris buffer saline (TBS) (RI = 1.333, viscosity = 0.7148 mPaS); detector oven temperature = 25°C. The system was calibrated with 500 µg (1.00 mg/ml) of conalbumin (75 kDa) or thyroglobulin (669 kDa) (Gel Filtration High Molecular Weight Calibration Kit, 28403842, GE Healthcare) dissolved in cage buffer. Parameters used for the standards were as follows: intrinsic viscosity (IV, dL/g) = 0; ratio between Weight Molecular Weight and Number Molecular Weight (M_W/M_N) = 1; M-H a = 0.7. Settings for calculation method used for assessing molecular weight included: calibration type – triple detection, analysis type – calculate dn/dc (or dA/dc) from sample concentration. Four measurements were performed for three different samples and the average was calculated.

***Mathematical Modelling* – prediction of small cage geometry.** To predict the structure of the small cage, the TRAP rings were modelled as regular hendecagons (11-sided polygons) and positioned on the vertices of regular polyhedra, i.e. Platonic and Archimedean solids, prisms and antiprisms. Not all regular polyhedra were used as we focused on those with 12 vertices. Pairs of hendecagons are joined edge-to-edge whenever the polyhedron's nodes are linked by an edge and all the possible edge-to-edge links are considered. As a result, potential cages built from 12 identical hendecagons are produced. The above-mentioned cage construction process is done automatically by a custom-written C++ program (of about 3,000 lines of code). For each polyhedral cage the program outputs a file describing the topology – i.e., all the links between the hendecagons.

A second C++ program (of around 8,400 lines of code) was then used to obtain the geometric attributes of the prospective cages. First, the hendecagonal faces were modelled as rigid bodies, with adjacent faces linked by two Hookean springs with a rest position set to 5% of the edge length. The energy of the system was then minimized via Monte Carlo algorithm. The coordinates of the resulting assembly were then used by the program to model the cage as a set of rigid rods using an energy functional divided into three terms: edge length deviations, face edge angular deviations, and degree of non-planarity. The first one measured by how much the edge lengths deviated from a chosen reference length. The second quantified by how much the angle between the hendecagon's edges deviated from the internal angle of the regular hendecagon. The third term computed the level of nonplanarity of the hendecagons. Each term was assigned a weight factor, with the planarity weight set to three orders of magnitude larger than for the lengths and the angles. This gave us structures with preserved planarity (with zero planarity distortions modulo numerical error). The energy functional was minimized using a Monte Carlo method and the program output a file containing the vertex coordinates, the topology of the cage, as well as the order of deformations obtained for the angles and edge lengths. The exact explanation of the algorithm will be given elsewhere but some details were already covered in.[7] From the polyhedral cages generated by the method described above, we selected the only candidate for which the dihedral angles and diameter were in line with the AFM measurements, and the deformation level was small (i.e. less than 2%). The modelled cage in **Figure 3** suggests a set of 48 S–Au–S bonds, one for each pair of joined hendecagonal vertices.

A small cage model with TRAP ring structures inserted into predicted polyhedron faces was built using Cage Builder tool of UCSF Chimera (version 1.14).[8]

Cryogenic electron microscopy (cryo-EM). Cryo-EM structure reconstruction was performed as described in **Figure S7**. Raw micrographs were motion corrected on the fly using Warp.[9] Motion corrected micrographs were imported to cryoSPARC and underwent further analysis. CTF estimation was performed by patchCTF job in cryoSPARC v2.15.0.[10] Examples of the statistics of the analyzed micrographs are shown in **Figure S8**. After CTF estimation, 1,000 particles were manually picked and classified in reference-free 2D classification in order to create templates for the automatic picking job (**Figure S7c-d**). Template-based picking gave 1,315,498 particles, which were again 2D classified and cleaned from bad particles. As a result, five 2D classes were selected with 671,845 particles **Figure S7f**). First, ab-initio reconstruction followed by homogenous refinement gave an initial 3D model with 5.93 Å resolution. This model was used to produced new, "artificial" 2D classes by back projection. 50 such classes were produced (**Figure S7g**) and used for a second round of template-based particle picking (2,006,399 particles were picked). Subsequent reference-free 2D classifications allowed us to sort particles and select the best 2D classes with 796,709 particles (**Figure S7j**). These particles were submitted to 3D classification resulting in three spherical particles with 2,928, 59,270 and 434,505 particles. Homogenous refinement of the best 3D class gave resolutions of 7.06 Å and 4.77 Å (after C_1 and T symmetry imposition respectively).

SUPPORTING FIGURES AND TABLES

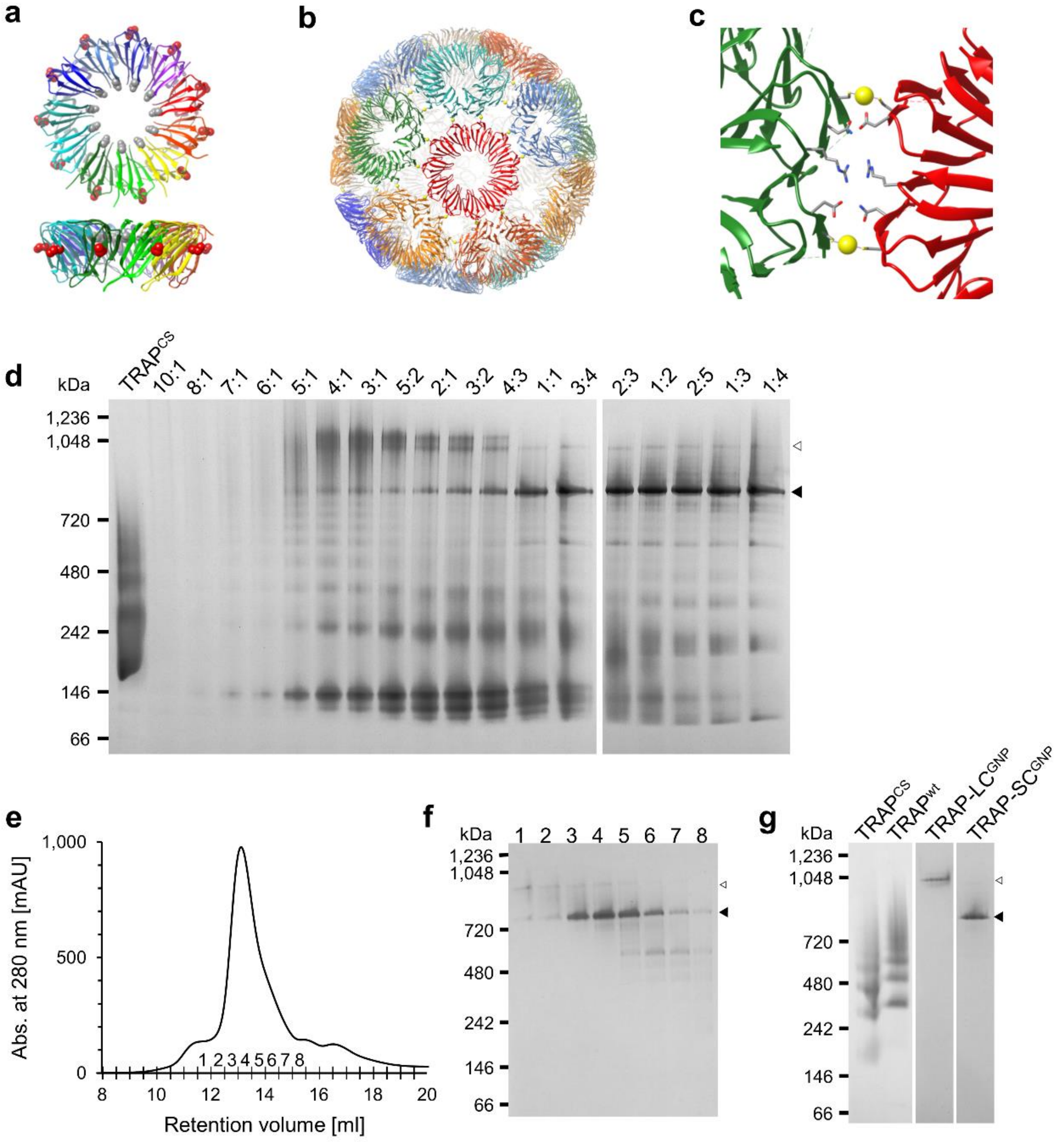

Figure S1. TRAP-SC^{GNP} production. (a-c) Known structure and features of the large (24-ring) TRAP-cage. (a) The structure of the TRAP ring (PDB: 1QAW)[11] shown in two orthogonal views with C35 as red spheres and S64 as grey spheres. (b) Structure of the left-handed large cage (PDB:

6RVV)[1] with gold atoms shown as yellow spheres. (c) A close up of rings interface with cysteines bridged via gold atom shown as a yellow sphere. (d) Native PAGE of TRAP(K35C/R64S) rings purified in absence of DTT incubated with GNPs for 3 days at room temperature. The lane marked "TRAP^{cs}" is without the addition of GNPs. In other cases the molar ratios of TRAP(K35C/R64S) monomer : GNPs are indicated. The hollow arrowhead indicates the position of TRAP-LC^{GNP}, the solid arrowhead the position of the TRAP-SC^{GNP}. (e) Size exclusion chromatogram (SEC) of 0.1 mM (monomer concentration) TRAP(K35C/R64S) after reaction with two molar excess (over TRAP monomer) of 1-3 nm gold nanoparticles (mAU, milli absorbance units). (f) Native PAGE confirms presence of small cage in fractions corresponding to the chromatogram (e). (g) Native PAGE of TRAP(K35C/R64S) rings (TRAP^{cs}), wild type TRAP rings (TRAP^{wt}), purified large cage (TRAP-LC^{GNP}) and purified small cage (TRAP-SC^{GNP}). On the gels the hollow arrowheads indicate the position of the large cage, the solid arrowheads – the position of the small cage.

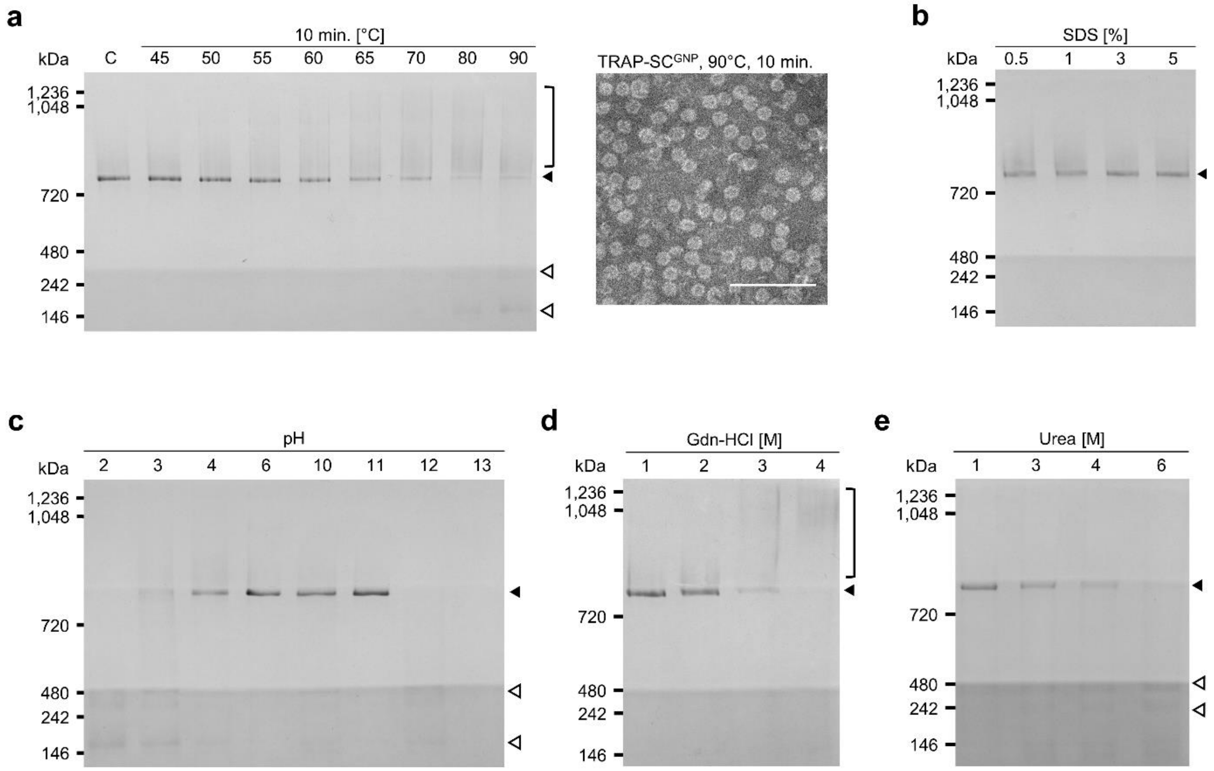

Figure S2. Stability of TRAP-SC^{GNP}. (a) Native PAGE gel (left) shows gradual destabilization of small cage, starting with aggregation at 55°C, followed by fragmentation at 80°C (C – small cage incubated at room temp.). The TEM image (right) was obtained after TRAP-SC^{GNP} incubation at 90°C for 10 min. (scale bar = 100 nm). (b) Influence of SDS on small cage stability. (c) Small cage is stable from pH 4–11 as shown by native PAGE. (d-e) Influence of guanidine hydrochloride (Gdn-HCl) and urea on small cage stability. On the gels, solid arrowheads indicate the position of small cage, hollow arrowheads – main disassembly bands, right bracket – smearing caused by aggregation.

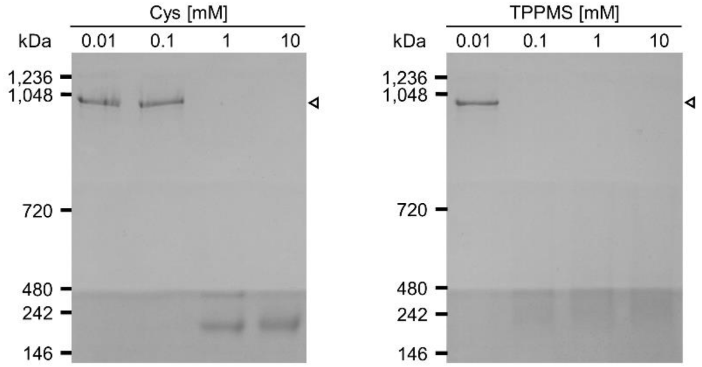

Figure S3. Controlled disassembly of TRAP-LC^{GNP} in presence of compounds containing thiol or phosphine groups: cysteine (Cys) and sodium salt of 3-(diphenylphosphino)benzenesulfonic acid (TPPMS). On the gels, arrowheads indicate the position of fully formed TRAP-LC^{GNP}.

a

Measurements	PDI	Mean deameter (nm)
1	0.026	17.78
2	0.080	18.27
3	0.050	18.44
4	0.024	17.91
5	0.019	18.10
6	0.014	17.91
7	0.003	18.22
8	0.010	18.37
Mean	0.014	18.13
S.D.	0.009	0.24

b

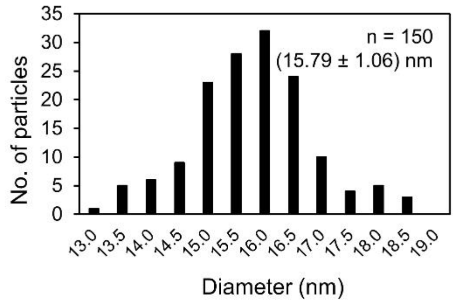

Diameter range	< 15 nm	15-17 nm	> 17 nm
Number of particles	29	105	16
(%)	19.3	70.0	10.7

c

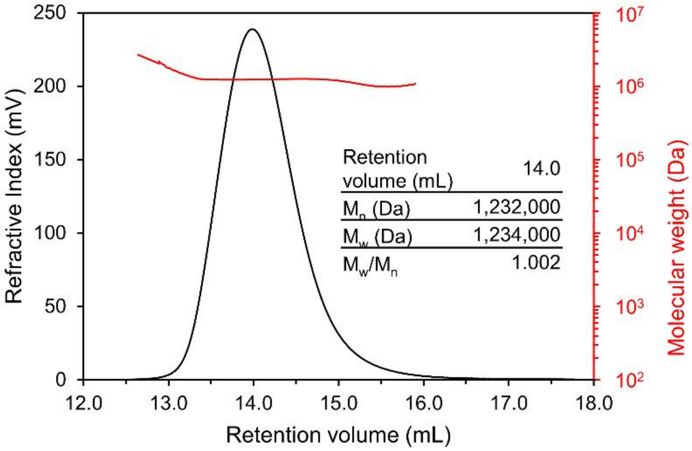

d

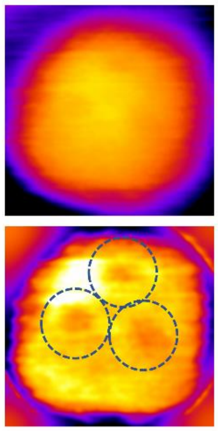

Figure S4. Details of TRAP-SC^{GNP} quaternary structure. (a) Average hydrodynamic diameter of TRAP-SC^{GNP} determined by dynamic light scattering (PDI, polydispersity index). The mean diameter is based on volume distribution. (b) Analysis of 150 particles from an electron micrograph of TRAP-SC^{GNP} (same sample as in Figure 1c) indicates mean diameter of around 16 nm. (c) Typical Refractive Index (RI) chromatogram of TRAP-SC^{GNP} with its measured molecular weight. Inset table shows retention volume, number (M_n) and weight (M_w) average molecular weights. M_w/M_n indicates polydispersity of the sample. (d) HS-AFM imaging of TRAP-SC^{GNP}(top) with three observable rings. Bottom panel shows the top image after the application of high-pass FFT frequency filter (10 nm).

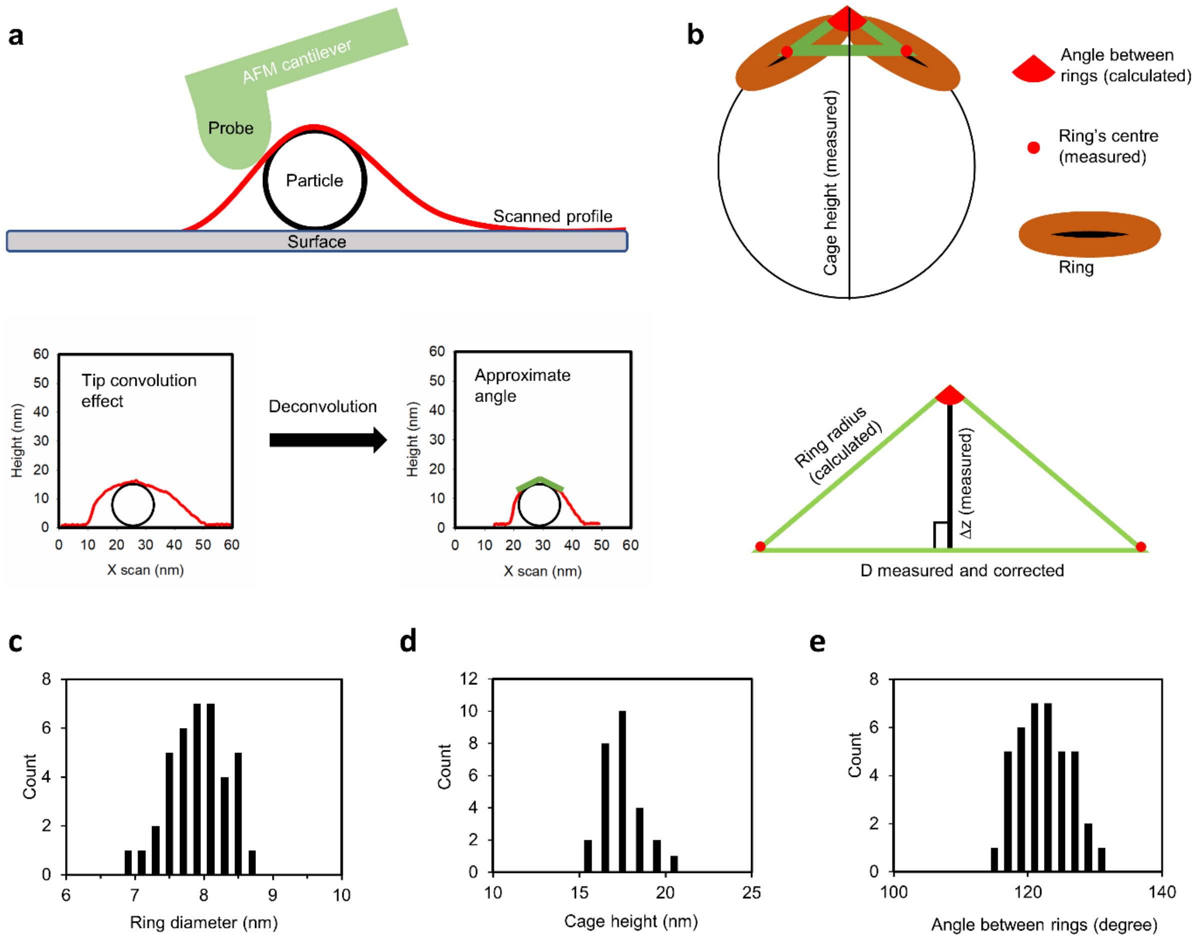

Figure S5. Principles behind AFM measurements. (a) Effect of the tip convolution on scanned profile. (b) Schematic representation of AFM measurement of cage height, angle between rings in cage and ring diameter. (c-e) Histograms of TRAP rings as measured by HS-AFM. (c) Vertical height profiles obtained from the measurement of 27 cages, resulting in a mean ring diameter of (7.9 ± 0.4) nm. (d) Cage height of (17.4 ± 1.1) nm and (e) angle between rings of (122.2 ± 3.8°).

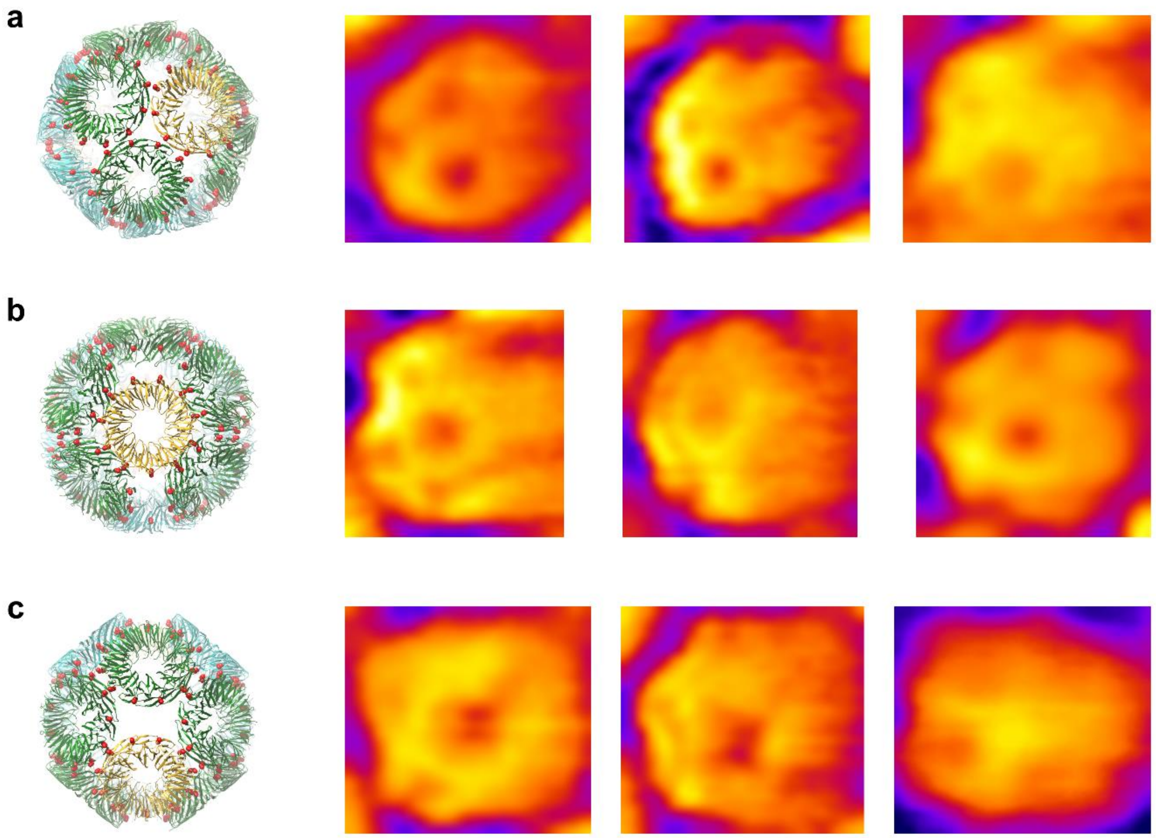

Figure S6. Examples of HS-AFM images: (right) of TRAP-SC^{GNP} showing three most frequently observed orientations on surface with the predicted possible small cage structure in corresponding orientation (left) centered at: (a) the 3-fold hole, (b) the TRAP ring, (c) the 2-fold hole.

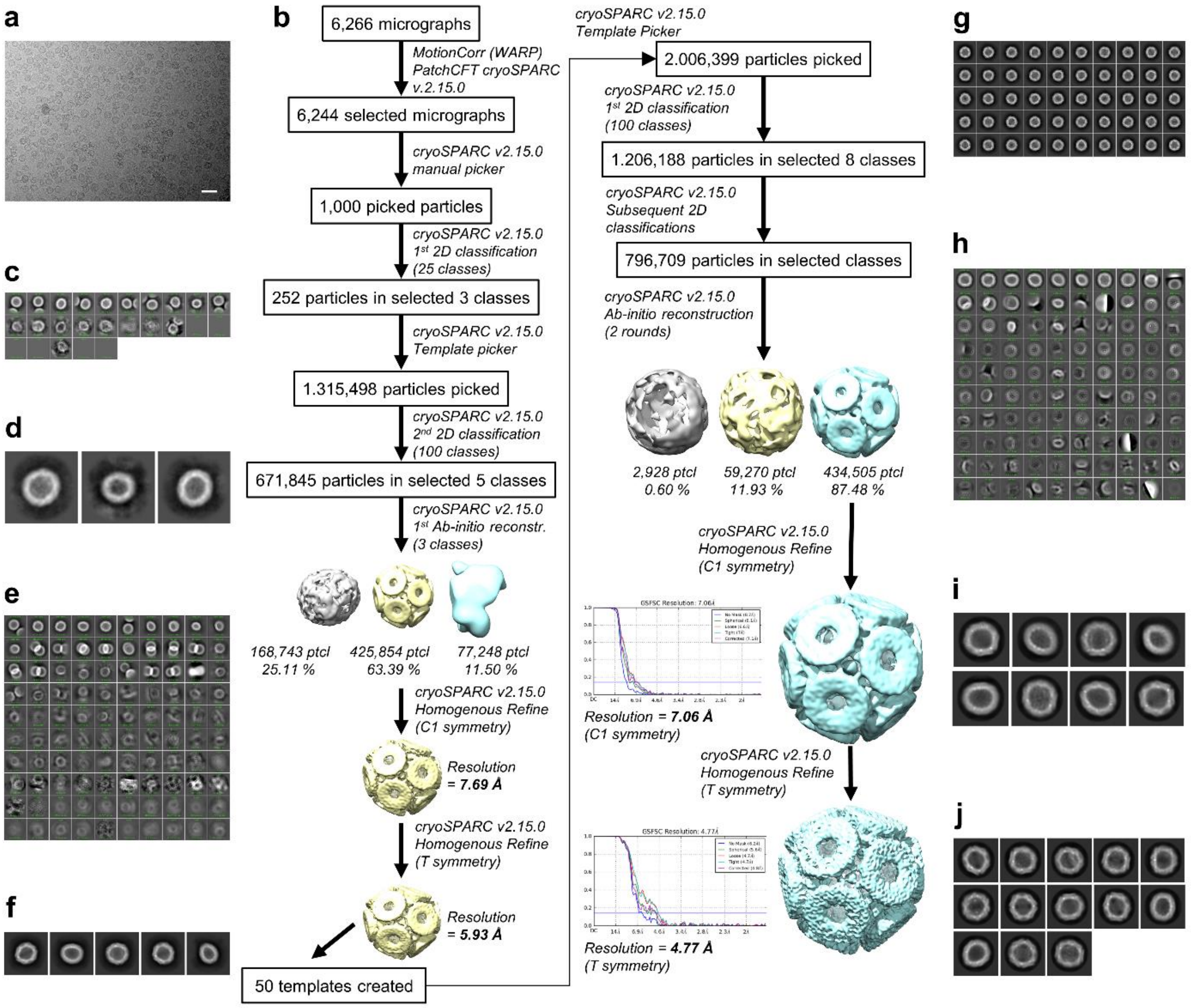

Figure S7. Procedure for cryo-EM reconstruction of TRAP-SC^{GNP} (EMD-12526): (a) Representative micrograph (scale bar = 50 nm). (b) Summary of the image processing procedure. (c) 2D class averages from reference-free 2D classification in cryoSPARC v2.15.0. (d) First selected three 2D classes. (e) Additional round of reference-free 2D classification. (f) Final 2D class averages from reference-free 2D classification in cryoSPARC v.2.15.0. (g) Artificial 2D projections of the first 3D model (50 different angular views). (h) Initial 100 reference-free 2D classes from template-picked particles. (i) Eight selected 2D class averages from reference-free

2D classification in cryoSPARC v2.15.0. (j) Final 2D class averages from reference-free 2D classification in cryoSPARC v.2.15.0.

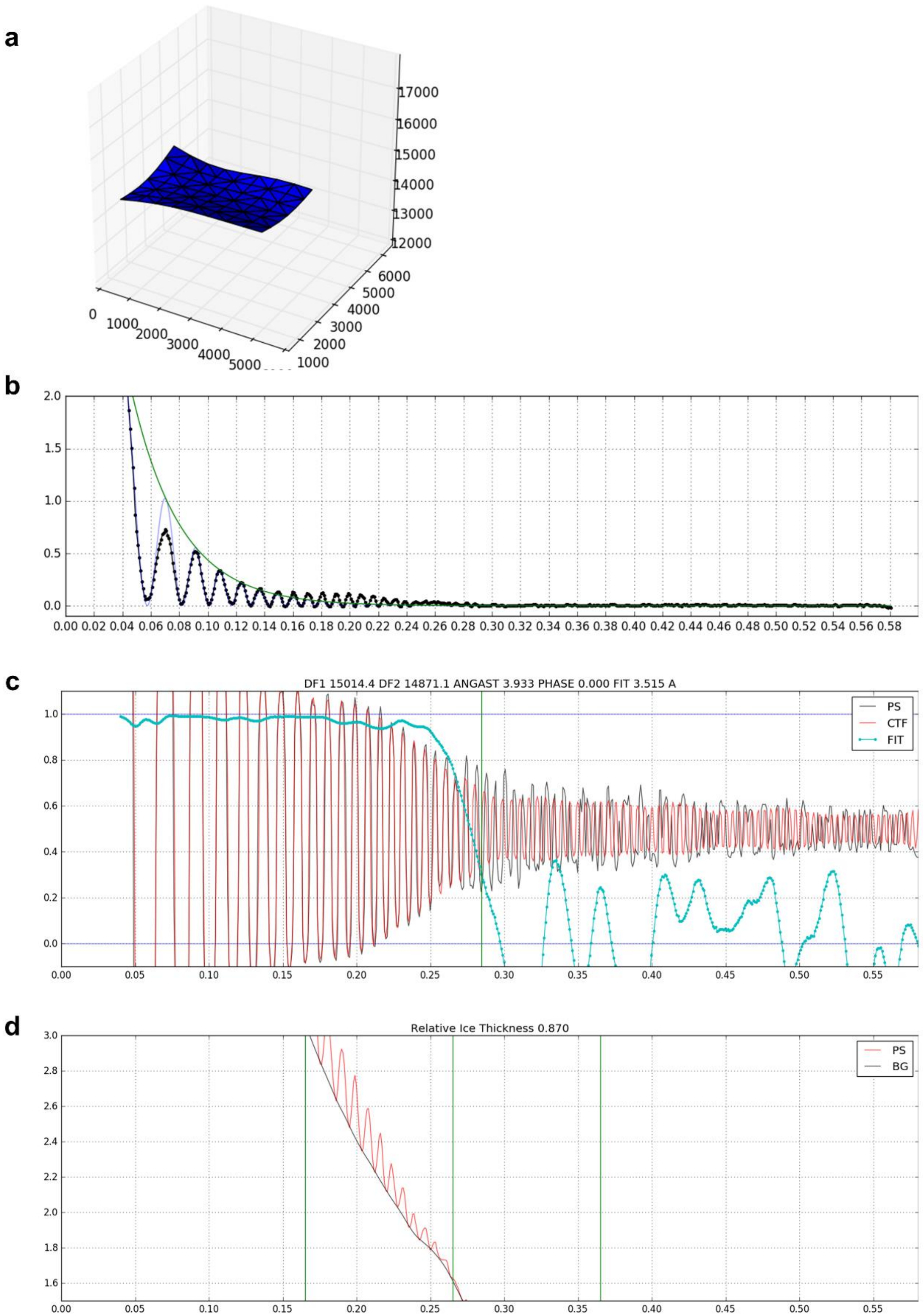

Figure S8. Example of the patch CTF calculation statistics: (a) 3D surface with local defocus estimation across the micrograph. (b) 1D CTF fit between simulated and observed Thon rings. (c)

Cross-correlation of the fit level of the CTF resolution (3.515 Å in this case). (d) Relative ice thickness plot (0.870 µm in this case).

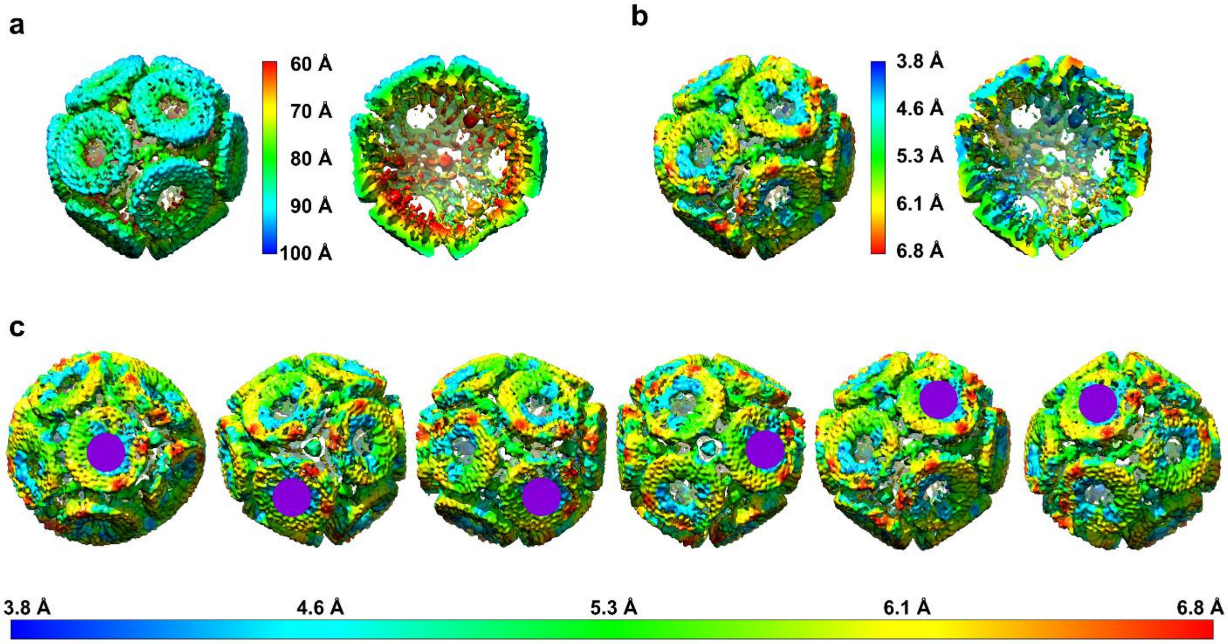

Figure S9. Different representations of the cryo-EM density. (a) Cryo-EM density coloured by distance from the center of the molecule – outside of the protein cage and its interior (left and right respectively). (b) Local resolution coloured cryo-EM density – outside of the protein cage and its interior (left and right respectively). (c) Local resolution coloured cryo-EM density focused on GNPs around one ring (marked with purple circle).

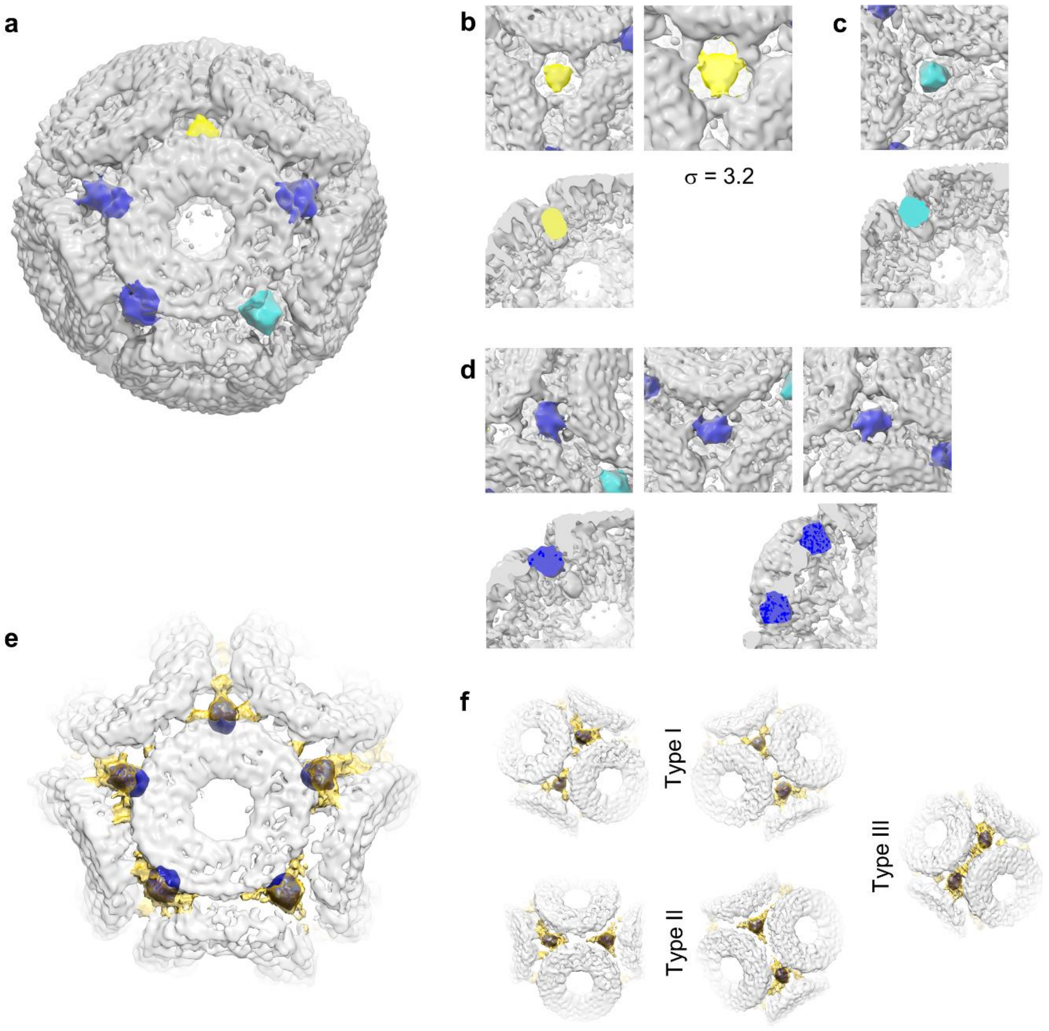

Figure S10. GNPs incorporation in the TRAP-SC^{GNP} structure: (a) Three types of GNPs inside TRAP-SC^{GNP} structure marked in different colours inside the overall density at σ = 3.5. (b) Type A GNP (yellow) that appears to have no direct interactions with protein part of the cage at σ = 3.5 (left) but clearly shows interaction at σ = 3.2 (right). (c) Type B GNP (cyan) surrounded by partial electron density suggesting that direct interactions with TRAP protein are possible and probably occurring. (d) Type C GNPs (blue) directly involved in interactions with protein parts of the TRAP small cage. In panels (b-d) top sub-panels show top view of the GNP, whereas bottom sub-panels show intersection of the density displaying the depth at which the gold nanoparticles are located. (e) Top view on one of the TRAP rings with marked GNPs (navy blue) and excessive electron density (gold). (f) Three types of connections with highlighted GNPs and additional electron densities. Overall volume of the additional densities is comparable to the overall volume

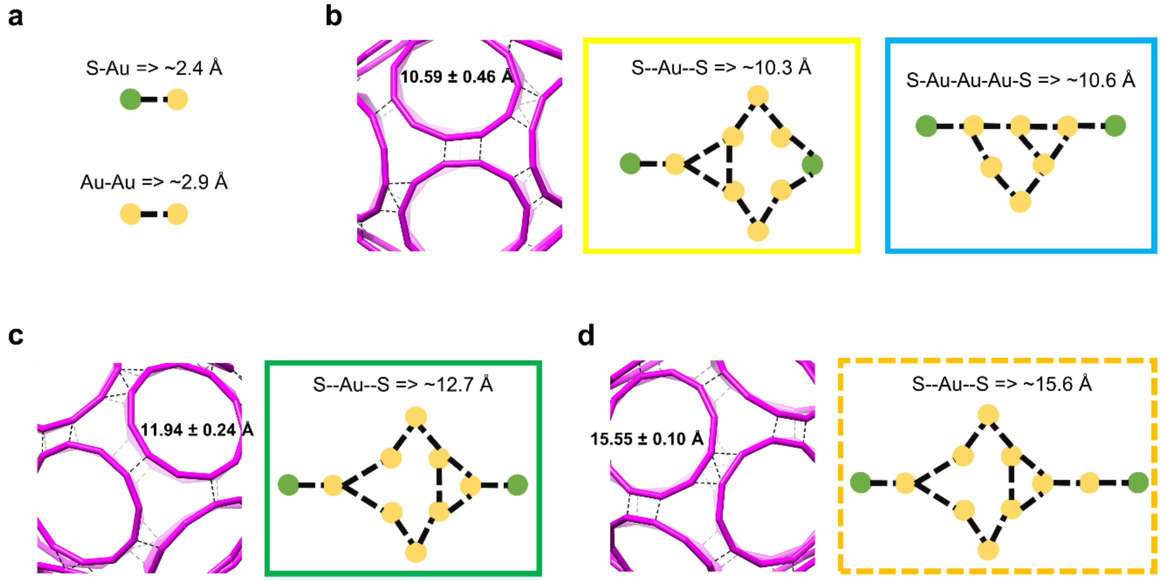

Figure S11. Types of connections and possible gold ions/atoms arrangements: (a) Average distances of the S-Au and Au-Au connections.[12] (b) Type I connection with measured S--Au--S distance (left) and calculated S--Au--S distances of possible gold "stitches" (right). (c) Type II connection with measured S--Au--S distance (left) and calculated S--Au--S distance of possible gold stich (right). (d) Type III connection with measured S--Au--S distance (left) and calculated S--Au--S distance of possible gold stich (right). Colours of solid frames are uniform around particular clusters. Dashed frame is an option not included in theoretical calculations, thus very probable to be present.

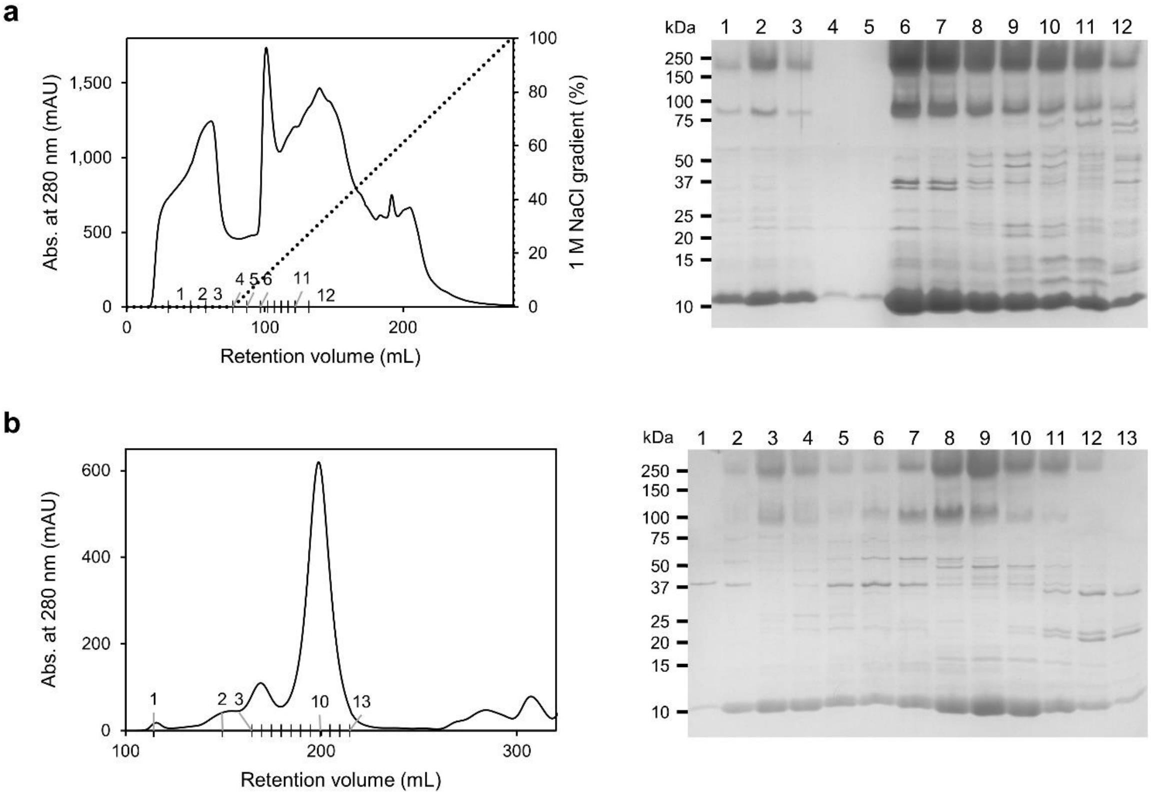

Figure S12. Depiction of chromatography steps in exemplary TRAP^{CS} purification procedure in absence of DTT. (a) Ion exchange chromatogram (left; 4 × 5 ml HiTrap QFF columns (GE Healthcare), binding in 50 mM Tris-HCl, pH 7.9, 0.05 M NaCl, eluting with a 0.05–1 M NaCl gradient) and corresponding denaturing gel (right). (b) Size exclusion chromatogram (left; HiLoad 26/600 Superdex 200pg column (GE Healthcare), eluting with 50 mM Tris-HCl, pH 7.9, 0.15 M NaCl) and corresponding denaturing gel (right).

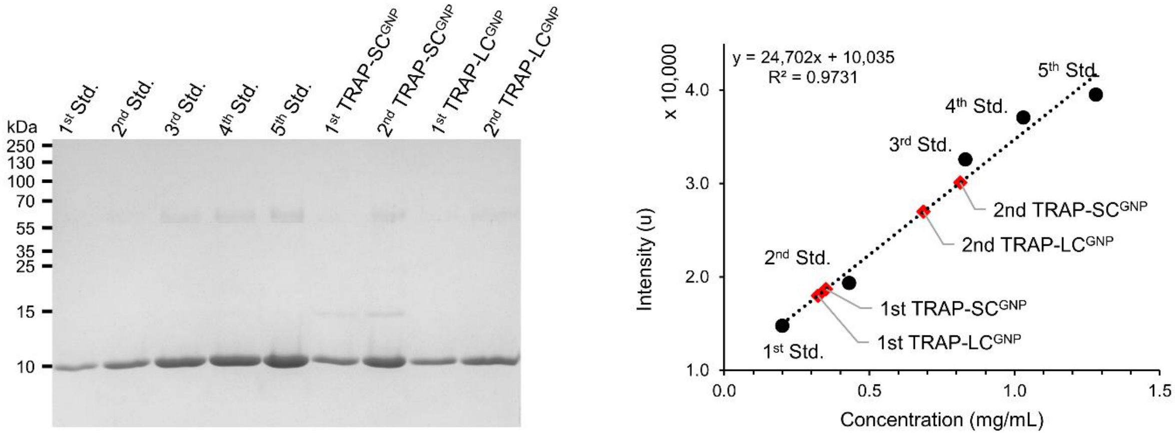

Figure S13. Example of GNP-induced large and small cage sample concentration estimation. Dilutions of unreacted TRAP(K35C/R64S) protein was used a standard and TRAP-LC^{GNP} and TRAP-SC^{GNP} samples were denatured and run on denaturing SDS gel (left). The resulting gel image was analysed in ImageJ software and a standard curve plotted based on bands intensities (right). Red dots correspond to calculated concentrations of the cages' samples.

Table S1. Comparison of stability GNP-induced large and small cage.

	TRAP-LC^{GNP} *	TRAP-SC^{GNP}
Temperature (°C), 10 min.	> 120	>90#
pH range	3-12	4-11
Urea (M)	> 7	3
Gnd-HCl (mM)	4	2
SDS (% (w/v))	> 3	> 3
DTT (mM)	0.1	0.7
TCEP (mM)	0.01	0.07
Glutathione reduced (mM)	1	0.7
L-Cysteine (mM)	0.1	0.1
TPPMS (mM)	0.01	0.01
Young modulus (MPa)	120	150

Numbers describing stability are the highest (lowest for the low pH) values where integrity of the cages was intact (no aggregation or cage disruption) as observed on native gels.

*Values for TRAP-LC^{GNP} based on results presented in reference[1] and **Figure 1, S2** and **S3**.

For TRAP-SC^{GNP} TEM results show majority of cages intact after 90°C incubation but aggregation at 50-60°C and some disassembly from 80°C and above, neither of which are significant for TRAP-LC^{GNP}.

Table S2. Table summarizing features of predicted structure of TRAP-SC^{GNP} (see also **Figure 3**).

Diameter:	16 nm
Dihedral angles:	
between two faces separated by a hole	101°
between adjacent faces	119°
Relative errors:	
edge length distortion	0%
angle distortion	1.83%
Holes:	
triangular	8
"bowtie"	6
Chirality:	NO
Rings are equivalent and each has 4 neighbours.	
Rotational symmetry:	
2-fold axis	3
3-fold axis	4
Underlying symmetry:	cuboctahedron

Table S3. Cryo-EM data collection statistics for TRAP-SC^{GNP} (see also **Figure S7**).

	**TRAP-SC^{GNP} (EMDB-12526)**
**Data collection and processing**	
**Magnification**	86000
**Voltage (kV)**	300
**Total exposure (e–/Å^2)**	40
**Defocus range (µm)**	-1.0 ÷ -3.5
**Pixel size (Å)**	0.86
**Symmetry imposed**	T
**Images collected (no.)**	6266
**Initial particles (no.)**	1.315,498
**Final particles (no.)**	434,505
**Map resolution (Å)**	4.77
**FSC threshold**	0.143

REFERENCES:

(1) Malay, A. D.; Miyazaki, N.; Biela, A.; Chakraborti, S.; Majsterkiewicz, K.; Stupka, I.; Kaplan, C. S.; Kowalczyk, A.; Piette, B. M. A. G.; Hochberg, G. K. A.; Wu, D.; Wrobel, T. P.; Fineberg, A.; Kushwah, M. S.; Kelemen, M.; Vavpetič, P.; Pelicon, P.; Kukura, P.; Benesch, J. L. P.; Iwasaki, K.; Heddle, J. G. An Ultra-Stable Gold-Coordinated Protein Cage Displaying Reversible Assembly. *Nature* **2019**, *569* (7756), 438–442. https://doi.org/10.1038/s41586-019-1185-4.

(2) Roos, W. H. How to Perform a Nanoindentation Experiment on a Virus. *Methods Mol. Biol.* **2011**, *783*, 251–264. https://doi.org/10.1007/978-1-61779-282-3_14.

(3) Snijder, J.; Uetrecht, C.; Rose, R. J.; Sanchez-Eugenia, R.; Marti, G. A.; Agirre, J.; Guérin, D. M. A.; Wuite, G. J. L.; Heck, A. J. R.; Roos, W. H. Probing the Biophysical Interplay between a Viral Genome and Its Capsid. *Nat. Chem.* **2013**, *5* (6), 502–509. https://doi.org/10.1038/nchem.1627.

(4) Maity, S.; Caillat, C.; Miguet, N.; Sulbaran, G.; Effantin, G.; Schoehn, G.; Roos, W. H.; Weissenhorn, W. VPS4 Triggers Constriction and Cleavage of ESCRT-III Helical Filaments. *Sci. Adv.* **2019**, *5* (4), eaau7198. https://doi.org/10.1126/sciadv.aau7198.

(5) Valbuena, A.; Maity, S.; Mateu, M. G.; Roos, W. H. Visualization of Single Molecules Building a Viral Capsid Protein Lattice through Stochastic Pathways. *ACS Nano* **2020**, *14* (7), 8724–8734. https://doi.org/10.1021/acsnano.0c03207.

(6) Imamura, M.; Uchihashi, T.; Ando, T.; Leifert, A.; Simon, U.; Malay, A. D.; Heddle, J. G. Probing Structural Dynamics of an Artificial Protein Cage Using High-Speed Atomic Force Microscopy. *Nano Lett.* **2015**, *15* (2), 1331–1335. https://doi.org/10.1021/nl5045617.

(7) Heddle, J. G.; Kowalczyk, A.; Piette, B. M. A. G. Hendecagonal Near-Miss Polyhedral

Cages. *Proceedings of Bridges 2019: Mathematics, Art, Music, Architecture, Education, Culture* **2019**, 363–366.

(8) Pettersen, E. F.; Goddard, T. D.; Huang, C. C.; Couch, G. S.; Greenblatt, D. M.; Meng, E. C.; Ferrin, T. E. UCSF Chimera - A Visualization System for Exploratory Research and Analysis. *J. Comput. Chem.* **2004**, *25* (13), 1605–1612. https://doi.org/10.1002/jcc.20084.

(9) Tegunov, D.; Cramer, P. Real-Time Cryo-Electron Microscopy Data Preprocessing with Warp. *Nat. Methods 2019 1611* **2019**, *16* (11), 1146–1152. https://doi.org/10.1038/S41592-019-0580-Y.

(10) Punjani, A.; Rubinstein, J. L.; Fleet, D. J.; Brubaker, M. A. CryoSPARC: Algorithms for Rapid Unsupervised Cryo-EM Structure Determination. *Nat. Methods* **2017**, *14* (3), 290–296. https://doi.org/10.1038/nmeth.4169.

(11) Antson, A. A.; Dodson, E. J.; Dodson, G.; Greaves, R. B.; Chen, X. P.; Gollnick, P. Structure of the Trp RNA-Binding Attenuation Protein, TRAP, Bound to RNA. *Nature* **1999**, *401* (6750), 235–242. https://doi.org/10.1038/45730.

(12) Zhao, Y.; Zhou, F.; Zhou, H.; Su, H. The Structural and Bonding Evolution in Cysteine-Gold Cluster Complexes. *Phys. Chem. Chem. Phys.* **2013**, *15* (5), 1690–1698. https://doi.org/10.1039/c2cp42830j.
